# Mathematical assessment of the role of temperature on desert locust population dynamics

**DOI:** 10.1371/journal.pone.0317040

**Published:** 2025-01-24

**Authors:** Dejen Ketema Mamo, Mathew N. Kinyanjui, Nourridine Siewe

**Affiliations:** 1 Department of Mathematics, Pan African University Institute for Basic Sciences, Technology, and Innovation, Nairobi, Kenya; 2 Department of Mathematics, Debre Berhan University, Debre Berhan, Ethiopia; 3 Department of Pure and Applied Mathematics, Jomo Kenyatta University of Agriculture and Technology, Nairobi, Kenya; 4 School of Mathematics and Statistics, College of Science, Rochester Institute of Technology, Rochester, New York, United States of America; Institute of Zoology Chinese Academy of Sciences, CHINA

## Abstract

This study presents a novel non-autonomous mathematical model to explore the intricate relationship between temperature and desert locust population dynamics, considering the influence of both solitarious and gregarious phases across all life stages. The model incorporates temperature-dependent parameters for key biological processes, including egg development, hopper growth, adult maturation, and reproduction. Theoretical analysis reveals the model’s capacity for complex dynamical behaviors, such as multiple stable states and backward bifurcations, suggesting the potential for sudden and unpredictable population shifts. Sensitivity analysis identifies temperature-related parameters as critical drivers of population fluctuations, highlighting the importance of accurate temperature predictions for effective management. Numerical simulations demonstrate the significant impact of temperature on population growth, with optimal conditions promoting rapid development and increased survival, while extreme temperatures can hinder population growth and trigger phase transitions. By providing a deeper understanding of temperature-driven population shifts, this model enhances the ability to predict locust outbreaks, optimize control strategies, and reduce the socio-economic and ecological impacts of locust invasions.

## 1 Introduction

Insect pests pose a significant threat to global food security, causing substantial yield losses and jeopardizing food availability [1, 2]. Among crops, the total global potential loss due to pests varied from about 50% in wheat to more than 80% in cotton production [3]. Among these pests, the desert locust (Schistocerca gregaria) stands out as a major menace to agricultural production worldwide [4]. Native to arid and semi-arid regions of Africa, the Middle East, and Asia [5], desert locusts have recently emerged as invasive threats in various regions, including South Asia and East Africa [6]. The life cycle of locusts encompasses three distinct stages: egg, hopper (nymph), and adult [7]. The duration of each stage varies due to ecological and weather conditions. Despite adverse conditions, solitarious locusts persist in desert regions, primed for mating once environmental conditions become favorable [8–10].

Pest dynamics are intricately linked to weather conditions, wherein temperature, humidity, and precipitation significantly influence pest population growth, development, and distribution [11, 12]. Temperature fluctuations can either accelerate or slow down pest life cycles, directly influencing reproductive rates and survival probabilities. For instance, warmer temperatures generally enhance metabolic rates, leading to accelerated development and heightened reproductive output in many insect pests [11, 12]. Conversely, extreme temperatures can induce physiological stress and elevate mortality rates. Humidity levels also play a pivotal role, as many pests thrive in moist environments conducive to their development and the propagation of plant diseases [13]. Precipitation patterns influence food and habitat availability, directly affecting pest population dynamics. Increased rainfall, for instance, can foster higher vegetation growth, thereby providing more resources for herbivorous pests, while drought conditions can curtail food supply and suppress pest populations [14, 15]. Advanced climate modeling and ecological forecasting emerge as indispensable tools for predicting pest outbreaks and implementing timely and effective pest management strategies in response to changing weather conditions [16, 17].

Desert locusts, one of the most notorious pests, exhibit population dynamics that are highly sensitive to weather conditions, with temperature playing a pivotal role in influencing various stages of their life cycle. Optimal temperature ranges are crucial for different developmental stages, impacting egg hatching, hopper development, and overall survival rates. For egg hatching, temperatures between 25°C to 35°C are ideal, facilitating rapid development and increased hatching success rates [18, 19]. Hopper development, the stage where locusts are in their immature, wingless form, thrives at temperatures ranging from 30°C to 40°C, with development rates accelerating significantly within this range [20, 21]. Adult locust morphology, including growth and reproduction, is most efficient at temperatures between 32°C and 38°C [22, 23]. Extreme temperatures, exceeding 40°C or falling below 20°C, can significantly increase mortality rates across all life stages, as a result of thermal stress and the disruption of key physiological processes [14, 24]. Thus, understanding and monitoring temperature variations in desert regions are crucial for predicting and managing locust outbreaks effectively.

Mathematical modeling stands as a cornerstone in elucidating the intricate dynamics of desert locust populations and their consequential impact. These models, employing differential equations and statistical methods, offer a quantitative lens into various biological phenomena, including locust population dynamics and disease spread [25, 26]. In the realm of mathematical biology, a particular emphasis is placed on comprehending population dynamics, crucial for informing strategies in ecology, conservation biology, and epidemiology [27, 28]. For insect pests like desert locusts, mathematical modeling becomes indispensable, facilitating simulations of population behaviors, influences of environmental factors, and the effectiveness of control measures [29, 30]. By incorporating climatic variables such as temperature, humidity, and wind conditions, these models predict breeding grounds, swarm locations, and potential crop damage, aiding in timely interventions and conservation efforts [31, 32]. Moreover, the integration of machine learning algorithms enhances forecasting accuracy and highlights regions at risk, underscoring the necessity for proactive monitoring and sustainable management practices [33, 34]. These modeling endeavors not only contribute to safeguarding agricultural resources and biodiversity but also hold promise in elucidating the complex interplay between locust dynamics and environmental changes, thus offering insights for future mitigation strategies [35, 36].

While previous research has significantly contributed to understanding desert locust population dynamics, there are notable gaps that our proposed model seeks to address. Existing models often oversimplify the complex transition between solitarious and gregarious phases, failing to capture the dynamic interactions driving these behavioral shifts. Our model introduces a novel approach to evaluating dual-phase dynamics, aiming for a more comprehensive understanding of locust population behavior [35]. While previous studies have considered the influence of temperature on egg hatching and hopper development, they often lack a precise mathematical representation of temperature variations and their effects on different life stages. These earlier works may have relied primarily on experimental observations without rigorous data fitting and model development, limiting their ability to quantitatively predict population dynamics under varying temperature conditions. Our model introduces detailed temperature-dependent equations for key life stages, including egg hatching, hopper development, and mortality rates, enhancing the predictive accuracy of locust population dynamics under diverse climatic conditions [28]. Furthermore, existing models overlook the impact of ecological factors on locust population dynamics, such as habitat suitability and resource availability [30]. Our model considers environmental carrying capacity limitations for eggs and hoppers, providing a more realistic portrayal of population growth constraints. Thus, the novelty of this work lies in its comprehensive approach to modeling desert locust population dynamics, addressing key gaps in the existing literature, and offering more accurate predictions for pest management and conservation efforts.

## 2 Models and methods

### 2.1 Model formulation

We present a new mathematical model to explore the population dynamics of desert locusts, a species characterized by solitarious and gregarious phases. The desert locust’s life cycle consists of three stages: eggs, nymphs/hoppers, and adults, with an average lifespan range from three to five months. Weather and climatic factors, particularly temperature, significantly influence the duration and success of each stage. The model divides the locust population at any time (*t*) into five compartments based on developmental stage and phase: eggs (*E*(*t*)), solitarious hoppers (*H*(*t*)), gregarious hopper/bands (*B*(*t*)), solitarious adults (*S*(*t*)), and gregarious adults/swarms (*G*(*t*)). These compartments facilitate tracking transitions between life stages and behavioral phases, offering a comprehensive representation of locust population dynamics.

The following key assumptions underlie the model formulation:

1. **Homogeneously mixed population:** The locust population is assumed to be uniformly distributed within the habitat, ensuring similar interaction rates among individuals.
2. **Fecundity based on female adults:** Egg production is directly proportional to the density of female adults, with a constant sex ratio unaffected by temperature variations.
3. **Logistic growth:** Egg deposition follows a logistic growth model.
4. **Phase transition:** The model accounts for transitions between gregarious (swarms) and solitarious phases during the adult stage.
5. **Oviposition rate:** Solitarious female adults lay eggs at a higher rate than their gregarious counterparts [37].
6. **Temperature dependence:** The rates of hatching and mortality for eggs are contingent upon the mean soil moisture levels, whereas the development and mortality rates of hoppers are affected by the average air temperature. It is assumed that other environmental factors are maintained at their optimal levels.
7. **Soil moisture and air temperature:** Under conditions of adequate rainfall, it is posited that the soil temperature at the depth of the pods and the air temperature exhibit analogous values, thereby permitting the use of air temperature as a dependable substitute for anticipating soil conditions critical to the development of eggs. [37].

The model variables, outlined in Table 1, serve as a foundation for the sequential construction of the population dynamics model. Desert locust eggs are laid by adult females in areas with bare sandy soil and require prior rainfall for successful incubation. The solitarious phase females have a greater egg-laying capacity compared to gregarious females [38]. This is reflected in the mathematical model’s equation for egg population dynamics

$$\begin{array}{c} \frac{dE}{dt}=\left(1-\eta \right)\phi \left(T\right)(1-\frac{E}{K})(S+\psi G)-(\sigma \left(T\right)+\mu_{e}\left(T\right))E, \end{array}$$ (1)

where *ϕ*(*T*) temperature-dependent egg-laying rate by adult females, *K* environmental carrying capacity for eggs, representing limitations due to moisture and suitable sandy soil for laying eggs, *η* is portion of adult male((1 − *η* is adult female), 0 < *ψ* < 1 is a factor to account for swarm lower egg-laying capacity compared to solitarious adults, *σ*(*T*) temperature-dependent egg hatching rate (1/*σ*(*T*) represents the average incubation period). The parameter *μ*_*e*_(*T*) temperature-influenced egg mortality rate.

**Table 1:** Description of variables.

Variables	Description
*E*(*t*)	Total number of eggs at time *t*
*H*(*t*)	Total number of solitarious hopper at time *t*
*B*(*t*)	Total number of gregarious hopper/band at time *t*
*S*(*t*)	Total number of solitarious adult at time *t*
*G*(*t*)	Total number of gregarious adult/ swarm at time *t*
*T*(*t*)	Mean soil or air temperature at time *t*

Hoppers emerge from the eggs and progress through several developmental stages (instars) before molting for the final time and becoming winged adults (fledglings). This development is temperature-dependent, accelerating at higher temperatures. The model incorporates a proportion (*θ*) of hatched eggs developing into solitarious hoppers, with the remaining (1 − *θ*) becoming bands. Two equations depict the population dynamics for both solitarious and gregarious hoppers.

$$\begin{array}{cc} \frac{dH}{dt} & =\theta \sigma \left(T\right)E-(\gamma_{h}\left(T\right)+\mu_{h}\left(T\right))H,\\ \frac{dB}{dt} & =\left(1-\theta \right)\sigma \left(T\right)E-(\gamma_{b}\left(T\right)+\mu_{b}\left(T\right))B, \end{array}$$ (2)

where *γ*_*h*_(*T*) and *μ*_*h*_(*T*) represent the rate of hopper development and mortality as a function of temperature, while *γ*_*b*_(*T*) and *μ*_*b*_(*T*) correspond to the temperature-dependent rates of band development and mortality.

Desert locusts go through a fascinating transformation as they change from young hoppers to adults. Both solitarious and gregarious hoppers have the potential to mature into adults, but the environment plays a crucial role in determining their final phase. When conditions are favorable, solitarious locusts can come together to form swarms (gregarization), while unfavorable conditions can cause gregarious swarms to break apart and return to the solitarious phase (dissociation). Additionally, if the conditions are suitable for one phase, individuals may remain in their current state (solitarious or gregarious) until the environment changes. This dynamic phase transition is represented by the equations describing the adult population dynamics of the model.

$$\begin{array}{cc} \frac{dS}{dt} & =\gamma_{h}\left(T\right)H+\beta_{2}G-\left(\beta_{1}+\mu_{s}\left(T\right)\right)S,\\ \frac{dG}{dt} & =\gamma_{b}\left(T\right)B+\beta_{1}S-\left(\beta_{2}+\mu_{g}\left(T\right)\right)G, \end{array}$$ (3)

where, *β*_1_ signifies the rate at which solitarious adults crowd to form swarms, whereas *β*_2_ refers to the rate at which swarms dissociate into solitarious phase adults. Furthermore, *μ*_*s*_(*T*) and *μ*_*g*_(*T*) represent the temperature-dependent natural mortality rates for solitarious and gregarious adults, respectively.

**Fig 1:**
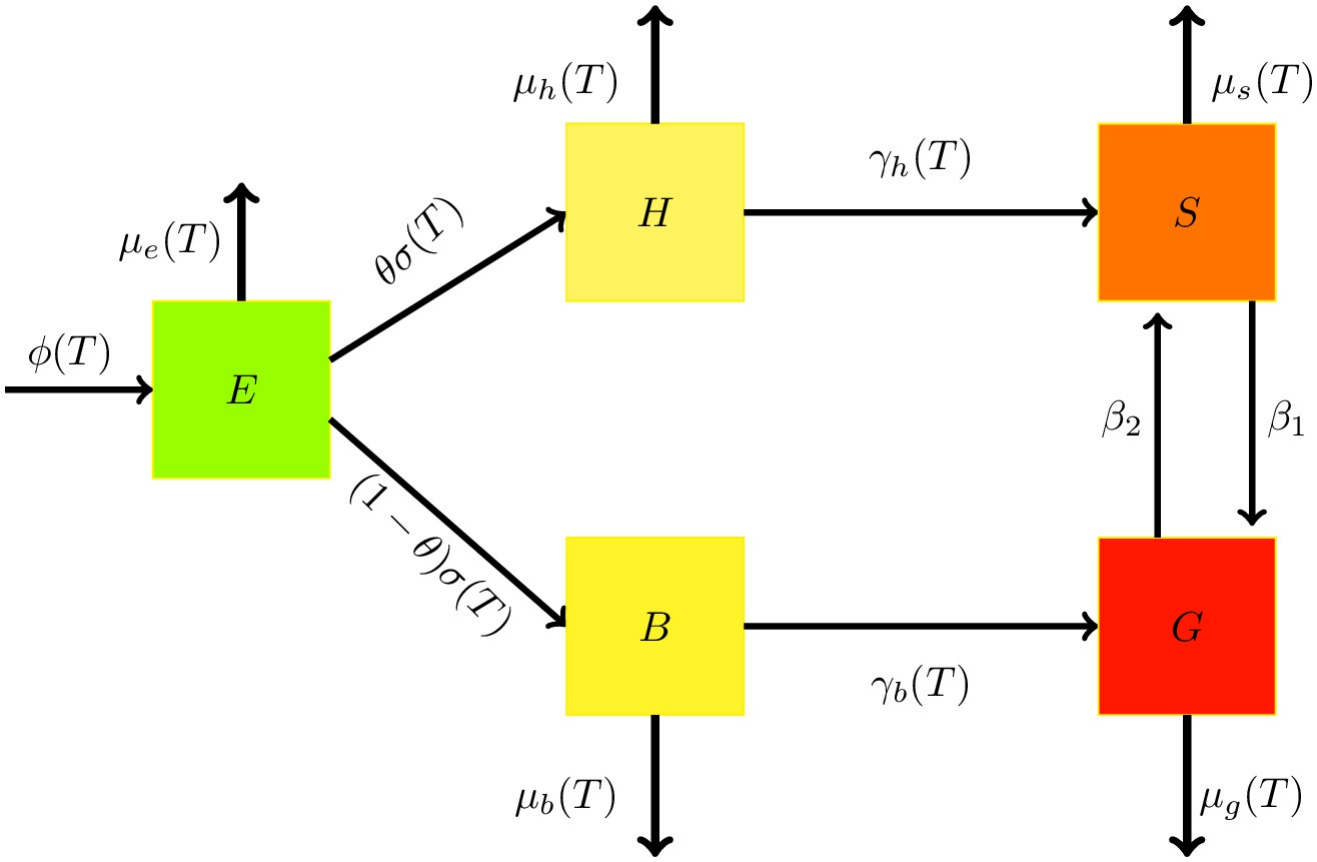
Desert locust dual phase population dynamics schematic diagram.

Based on the assumptions outlined previously, the conceptual diagram in Fig 1, and the Eqs [1–3] the population dynamics of desert locusts are modeled using a non-autonomous system of nonlinear differential equations:

$$\begin{array}{cc} \frac{dE}{dt} & =\left(1-\eta \right)\phi \left(T\right)(1-\frac{E}{K})(S+\psi G)-(\sigma \left(T\right)+\mu_{e}\left(T\right))E,\\ \frac{dH}{dt} & =\theta \sigma \left(T\right)E-(\gamma_{h}\left(T\right)+\mu_{h}\left(T\right))H,\\ \frac{dB}{dt} & =\left(1-\theta \right)\sigma \left(T\right)E-(\gamma_{b}\left(T\right)+\mu_{b}\left(T\right))B,\\ \frac{dS}{dt} & =\gamma_{h}\left(T\right)H+\beta_{2}G-\left(\beta_{1}+\mu_{s}\left(T\right)\right)S,\\ \frac{dG}{dt} & =\gamma_{b}\left(T\right)B+\beta_{1}S-\left(\beta_{2}+\mu_{g}\left(T\right)\right)G. \end{array}$$ (4)

The model starts with non-negative initial population values for all compartments:

$$\begin{array}{c} E(0)\geq 0,\;H(0)\geq 0,\;B(0)\geq 0,\;S(0)\geq 0,\;G(0)\geq 0. \end{array}$$ (5)

For simulation purposes, a generalized temperature function [39] given by

$$\begin{array}{c} T\left(t\right)=T_{0}[1+T_{1}\;\text{cos}\;(\frac{2\pi }{365}\left(\omega t+\kappa \right))], \end{array}$$ (6)

where *T*_0_ is the mean annual temperature, *T*_1_ represents the variation about the mean, *ω* measures the periodicity of the function and *κ* is the phase shift of the function. The Therefore the time-dependent temperature *T* = *T*(*t*), the temperature-dependent parameters *ϕ*(*T*), *σ*(*T*), *γ*(*T*), *μ*_*e*_(*T*), *μ*_*h*_(*T*) and *μ*_*b*_(*T*) are bounded, positive and *ω*-periodic functions. That is they belong to $L_{+}^{\infty }(0,\omega ,{\mathbb{R}}_{+})$.

### 2.2 Parameter selection

The derivation of the fundamental values for the established parameters of the model (4) is delineated as follows: Oviposition by female locusts occurs in association with the likelihood of encountering adult male locusts *η* and adult female locusts (1 − *η*). According to data collected from surveys, the mean sex ratio is expressed as the ratio of the number of females to the sum of the number of females and males. The percentage range of females spans from 49.9 to 69.8 [40], thereby indicating that the percentage of males is 100 minus the female percentage. Consequently, the probability of males, *η*, based on the sex ratio, is 0.302 to 0.501. The value of the daily fecundity rate parameter *ϕ* is derived from a study concerning the population parameters of desert locusts [37, 41], by considering the per capita fecundity rate of female adult locusts. Considering that a desert locust’s lifespan ranges from three to five months [42], the number of eggs per generation is 400 for solitarious females and 140 for gregarious females [37], leading to the determination of the modification parameter *ψ* = 140/400 = 0.35. The fecundity rate, *ϕ*, for gregarious locusts ranges from $\frac{140}{\left(5\times 30\right)}=0.9333$ to $\frac{140}{\left(3\times 30\right)}=1.5556$, while for solitarious locusts it ranges from $\frac{400}{\left(5\times 30\right)}=2.6667$ to $\frac{400}{\left(3\times 30\right)}=4.4444$. Eggs are typically deposited in regions characterized by exposed sandy soil. Generally, oviposition by females does not occur unless the soil exhibits moisture at a depth of approximately 5–10 cm beneath the surface [37]. Estimates of egg pod densities vary from 200–500/m^2^ [38]. During the gregarious phase, Desert Locusts produce pods containing fewer than 80 eggs, while in the solitarious phase, the pods customarily contain between 90 and 160 eggs [37]. Taking into account the maximum egg count per pod as 160 and utilizing the maximum pod density per square meter, the upper bound for the environmental carrying capacity of eggs per square meter is *K* = 160 × 500 = 80, 000 eggs per square mater. The incubation period is defined by 1/*σ*, with eggs estimated to hatch over an average duration of two weeks, depending on temperature (varying from 10 to 65 days), resulting in 1/*σ* for 10 to 65 days, thus the hatching rate *σ* range form 0.0154 to 0.1 on average *σ* = 0.0714) [37, 42]. Estimates of egg mortality indicate total losses varying from 5% to 65%, with average losses posited to be about 13% in solitarious populations and about 33% in gregarious populations (therefore, *μ*_*e*_ range from 0.05 to 0.65).

During the hatching phase, the nascent hoppers navigate through the froth plug to reach the surface. They promptly undergo a moult to the first instar and progress through five instars (occasionally six in the solitary phase) [38]. The developmental duration for solitarious nymphs, as defined by 1/*γ*_*h*_, ranges from 30–48 days, thereby corresponding to a developmental rate *σ*_*h*_ range from 0.0208 to 0.0333. Analogously, the developmental period for gregarious nymphs, as established by 1/*γ*_*b*_, spans 25 to 57 days, leading to a development rate *γ*_*b*_ range form 0.0175 to 0.04 [37]. Field data indicate mortality rates of 70% for the first instar, 20% for the second instar, 10% for the third to fifth instars, and 5% at each moult [37, 38]. The overall mortality computation for nymphal bands is deduced from a specific formula, where *M*_*i*_ represents the mortality rate of a given instar *i* and *S*_*i*_ = 1 − *M*_*i*_ signifies the survival rate for that instar *i*. Accordingly, survival probabilities *S*_1_ = 1 − 0.7 = 0.3, *S*_2_ = 1 − 0.2 = 0.8, *S*_3_ = *S*_4_ = *S*_5_ = 1 − 0.1 = 0.9, are calculated per moult *S*_*m*_ = 1 − 0.05 = 0.95. The overall probability of survival is calculated as the multiplicative product of survival probabilities across each developmental stage and moult transitions. The Total Survival Probability is computed as the product of survival probabilities across all stages and moults, encompassing four moults (one between each instar): $S_{\text{total}}=S_{m}^{4}\prod_{i=1}^{4}S_{i}$. The aggregate mortality rate is *μ*_*b*_ = 1 − *S*_total_ = 0.8575. Notably, there is a variation of merely one moult stage concerning solitarious hopper mortality, thus *μ*_*h*_ = *μ*_*b*_ × 0.95 × 0.9 = 0.7332. The lifespan of desert locusts, irrespective of being solitarious or gregarious, ranges from 90 to 150 days [42]. Consequently, the mortality rate for adult solitarious *μ*_*s*_ and gregarious *μ*_*g*_ is a range from 1/150 = 0.0067 to 1/90 = 0.0111.

## 3 Results

### 3.1 Temperature-dependent parameters

This subsection explores the influence of temperature on key model parameters. To quantify these relationships, nonlinear least squares regression is employed using MATLAB’s lsqcurvefit function.

#### 3.1.1 Egg oviposition rate

The average number of eggs laid by a female desert locust varies with temperature [45, 46]. Optimal egg-laying activity occurs between 25°C and 35°C. In this range, females exhibit higher egg production throughout their lifespan. However, temperatures below 20°C or above 35°C can hinder reproduction, leading to lower egg-laying rates [46]. Extremely high (> 40°C) or low (< 20°C) temperatures can significantly reduce egg production. While warmer temperatures generally promote increased reproduction, exceeding certain thresholds significantly inhibits egg-laying, impacting the average number of eggs per female [46, 47]. The dependence of fecundity on temperature is effectively characterized by a Gaussian function [48]:

$$\begin{array}{c} \phi \left(T\right)=E_{\text{max}}\;\text{exp}\;(-{\left(\frac{T-T_{\text{opt}}}{T_{w}}\right)}^{2}), \end{array}$$ (7)

where *E*_max_ denotes the maximum fecundity rate achieved at the optimal temperature across the lifespan, *T*_opt_ refers to the temperature at which this optimal egg production occurs, and *T*_*w*_ represents the parameter that defines the breadth of the temperature range within which optimal egg-laying is facilitated.

**Fig 2:**
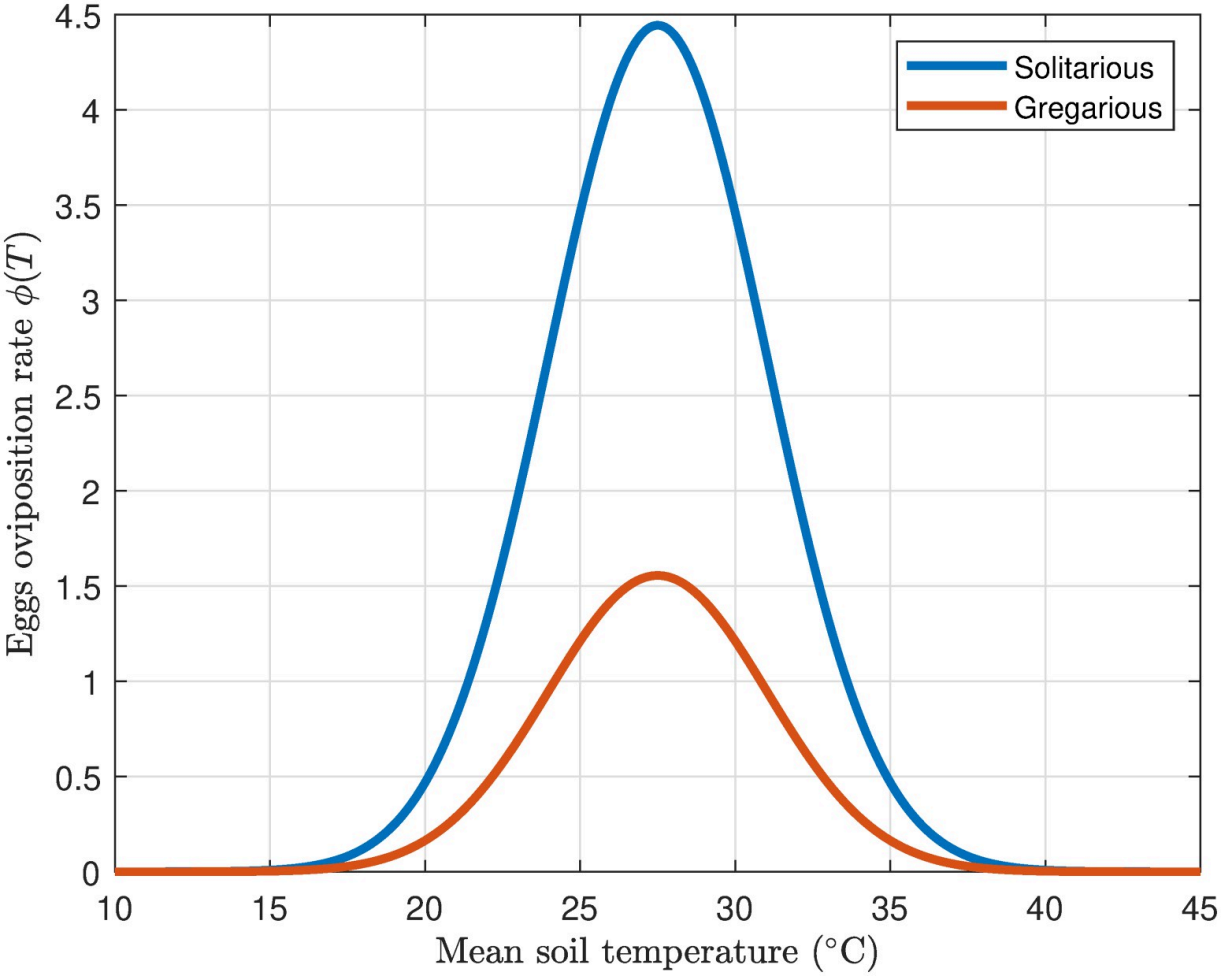
Profile of temperature-dependent oviposition rate of adult desert locusts using the function (7).

Fig 2 demonstrates the simulated association between temperature, measured in Celsius, and the fecundity rate of solitarious and gregarious desert locusts. The simulation employed model parameters, including an optimal temperature of 30°C, a temperature width parameter of (*T*_*w*_) valued at 8°C, and the maximum fecundity rate, as detailed in section 2.2, for solitarious (*E*_max_) = 1.5556 and gregarious (*E*_max_) = 4.4444 locusts. As anticipated, the figure corroborates that solitarious adult locusts exhibit a higher rate of egg production relative to their gregarious counterparts, thereby supporting assumption 2 and aligning with the findings of [49].

#### 3.1.2 Egg hatching rate

The Desert Locust primarily deposits its eggs in soil that possesses sufficient moisture to enable the absorption essential for the completion of development. Therefore, the developmental rate is solely contingent upon the soil temperature at the depth of the egg pod, taking into account the presence of adequate rainfall. A substantial correlation exists between soil and air (screen) temperatures, facilitating an accurate prediction of egg developmental rates based on air temperature measurements [37]. The success of desert locust egg hatching is markedly influenced by daily soil temperature. Optimal hatching is achieved within a temperature range of 25°C to 35°C, with eggs customarily hatching within two weeks under these conditions [38]. In contrast, lower temperatures can notably prolong the incubation period, with hatching times extending from 10 to 65 days. Conversely, excessively high temperatures may diminish egg viability or entirely impede hatching [50]. The pivotal role of temperature in regulating egg hatching highlights its substantial influence on the population dynamics of desert locusts.

To model the temperature-dependent egg incubation period, the Allahyari equation is employed [51]:

$$\begin{array}{c} \sigma \left(T\right)=P{\left(\frac{T-T_{\text{min}}}{T_{\text{max}}-T_{\text{min}}}\right)}^{n}\times (1-{\left(\frac{T-T_{\text{min}}}{T_{\text{max}}-T_{\text{min}}}\right)}^{m}) \end{array}$$ (8)

where *P* is a scaling factor, *n* and *m* are exponents controlling the shape of the curve *T*_min_ and *T*_max_ are the minimum and maximum lethal temperatures.

**Fig 3:**
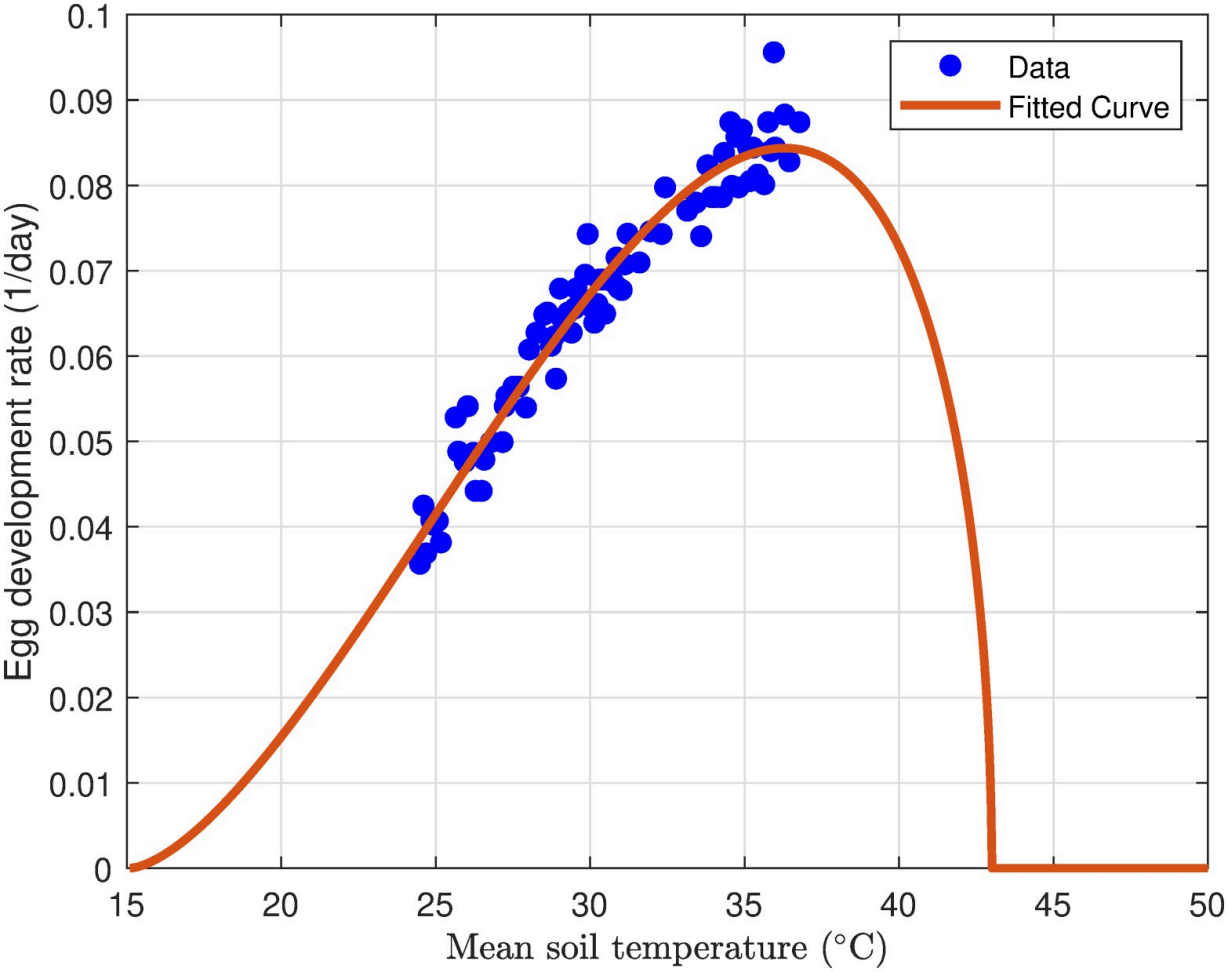
Profile of temperature-dependent desert locust egg hatching rate (1/days).

The parameters were estimated using temperature-versus-hatching day data from [38]. Fig 3 illustrates the simulated egg development rate (1/days) as a function of mean soil temperature. The fitted curve based on the function (8) captures the overall trend of temperature-dependent egg development. The estimated parameter values are: *P* = 0.2658, *n* = 1.5818, *m* = 0.5, *T*_min_ = 15.1°C, and *T*_max_ = 42.9187°C.

As shown in Fig 3, egg development becomes inviable below 15.1°C and 42.9187°C. These findings indicate that desert locust egg development is strongly influenced by temperature, with increasing hatching occurring between 25°C and 35°C. Lower temperatures extend the incubation period, while higher temperatures can inhibit or prevent hatching. The estimated lethal temperature thresholds highlight the critical role of temperature in regulating desert locust population dynamics. The curve captures the nonlinear relationship between temperature and hatching rate, with a gradual increase in hatching rates as temperature approaches the optimum and a sharp decline at temperature extremes.

#### 3.1.3 Egg mortality rate

Desert locust egg survival is significantly influenced by temperature. Optimal development occurs within a temperature range of 25°C and 35°C. Temperatures below 10–15°C can halt development, while prolonged exposure to temperatures exceeding 40°C during active growth stages is lethal [52]. Additionally, high soil temperatures around 35°C can increase egg mortality rates. Overall egg loss, encompassing factors such as inviability, failed hatching, predation, and environmental conditions, can range from 5% to 65%. solitarious locust populations typically experience lower egg losses (around 13%) compared to gregarious populations (around 33%) [37].

To model the temperature-dependent egg mortality rate, the following equation is used [53]:

$$\begin{array}{c} \mu_{e}\left(T\right)=1-\frac{M}{\text{exp}\;((1+e^{\left(\frac{T_{\text{min}}-T}{Q}\right)})\times (1+e^{\left(\frac{T-T_{\text{max}}}{Q}\right)}))}, \end{array}$$ (9)

where *μ*_*e*_(*T*) is the desert locust egg mortality rate at temperature *T*(°C), *T*_min_ is the minimum lethal temperature, *T*_max_ is maximum lethal temperature, while *M* and *Q* are constant values of model parameters.

**Fig 4:**
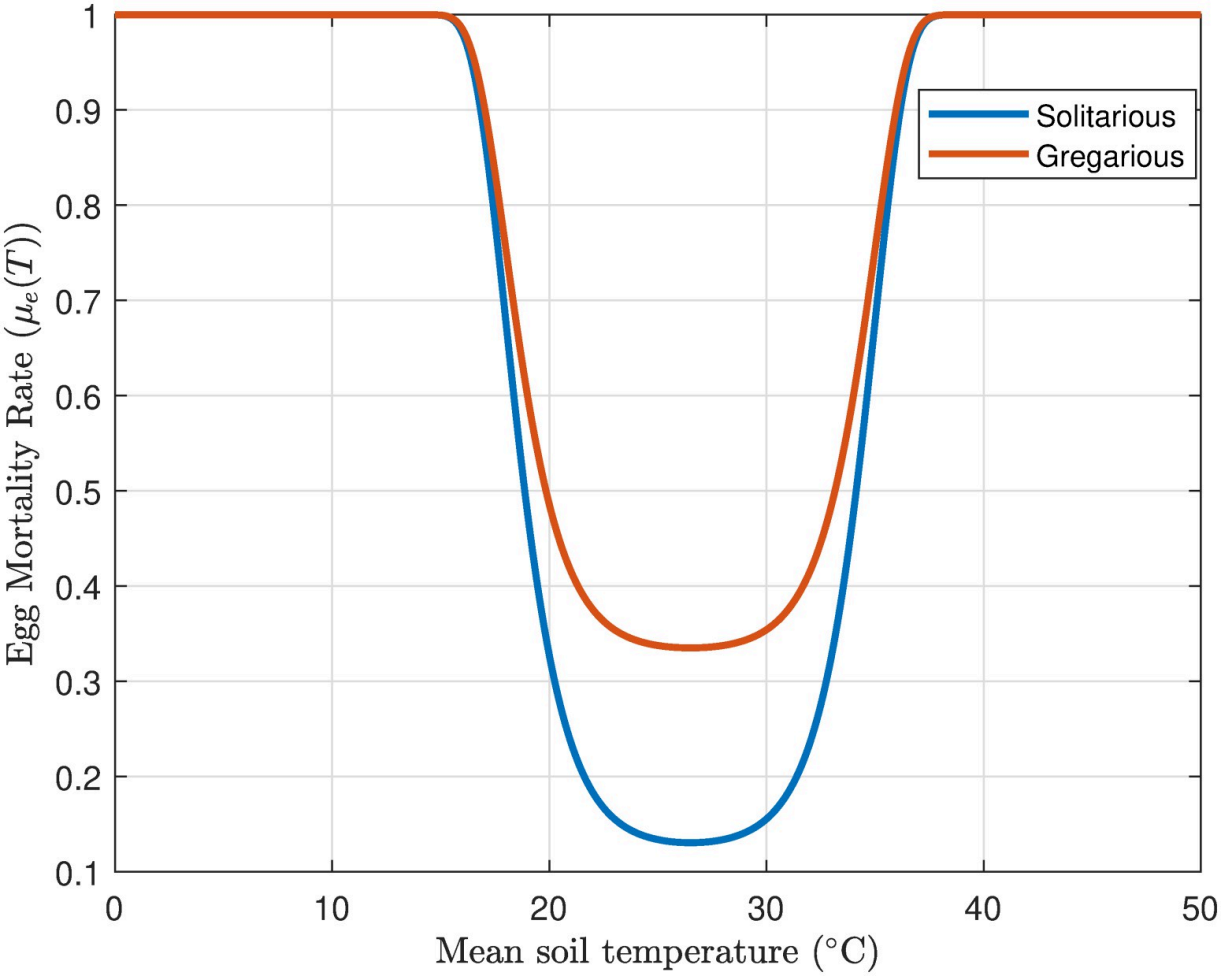
Profile of egg mortality rate of desert locusts as a function of soil temperature (°C).

Fig 4 depicts the proportion of egg mortality rate as a function of soil temperature. The model anticipates a non-linear relationship between temperature and egg mortality, with mortality rates accentuating at extreme temperatures. The optimal temperature range for minimal egg mortality is situated between 18°C and 35°C. The model indicates that gregarious locusts suffer from elevated egg mortality rates at (*M* = 0.91°C) compared to solitarious locusts at (*M* = 1.19°C) under analogous temperature conditions and *Q* = 1.5°C.

#### 3.1.4 Hopper development rate

The development rate of desert locust hoppers is strongly influenced by temperature. Optimal development occurs around 35°C, where hoppers progress rapidly through their life stages. Temperatures below 15°C significantly slow down metabolic processes, extending development time and increasing vulnerability. While hoppers can tolerate temperatures up to 45°C, prolonged exposure leads to physiological stress, reduced growth, and increased mortality [54]. Studies by [5, 38, 50] provide detailed insights into the ecological and environmental factors affecting locust development. These findings highlight the critical role of temperature in locust population dynamics. Notably, desert locust hoppers and adults thrive within a temperature range of 23°C and 40°C [52].

To model the temperature-dependent development of desert locust hoppers or bands, the Allahyari Eq 8 was used.

**Fig 5:**
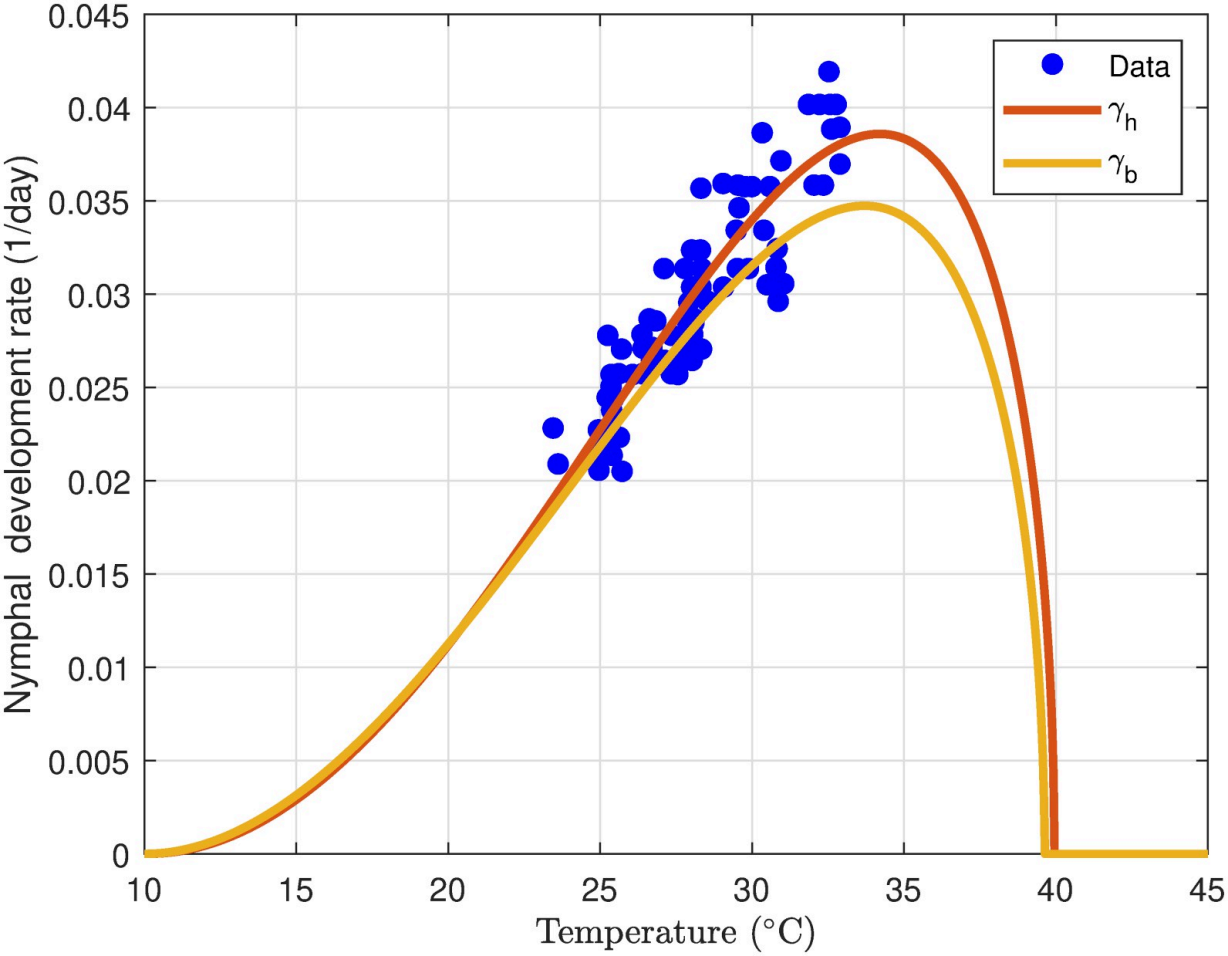
Profile of the relationship between air temperature (°C) versus desert locust hopper development rate (1/day).

Fig 5 illustrates the simulated development rate of desert locust hoppers as a function of air temperature, based on data from [42]. The fitted curve, derived from Eq (8), has parameters *P* = 0.1192, *n* = 1.3176, *m* = 0.5, *T*_*min*_ = 15°*C*, *T*_max_ = 45.012°C and captures the full range of temperatures influencing the time taken for hoppers to progress through their five instar stages. The model suggests that hopper development accelerates with increasing temperature within an optimal range, but extreme temperatures, both high and low, can significantly delay development or even cause mortality. The estimated parameters indicate a minimum threshold for development at 15°C and a maximum tolerance of approximately 45°C. These findings corroborate previous research on locust phenology by [38, 50]. The developmental rate of hoppers, akin to that of eggs, is contingent upon temperature. However, the association with ambient air temperatures is less pronounced in hoppers compared to eggs, as hoppers possess the ability to regulate their body temperature significantly by engaging in behaviors such as basking or seeking refuge in shade [37].

#### 3.1.5 Hopper mortality rate

Desert locust hoppers are highly susceptible to temperature extremes. Temperatures below 15°C slow metabolic processes, extending development times and increasing mortality risk. Conversely, while hoppers can tolerate temperatures up to 45°C, prolonged exposure leads to physiological stress, reduced growth, and increased mortality.

**Fig 6:**
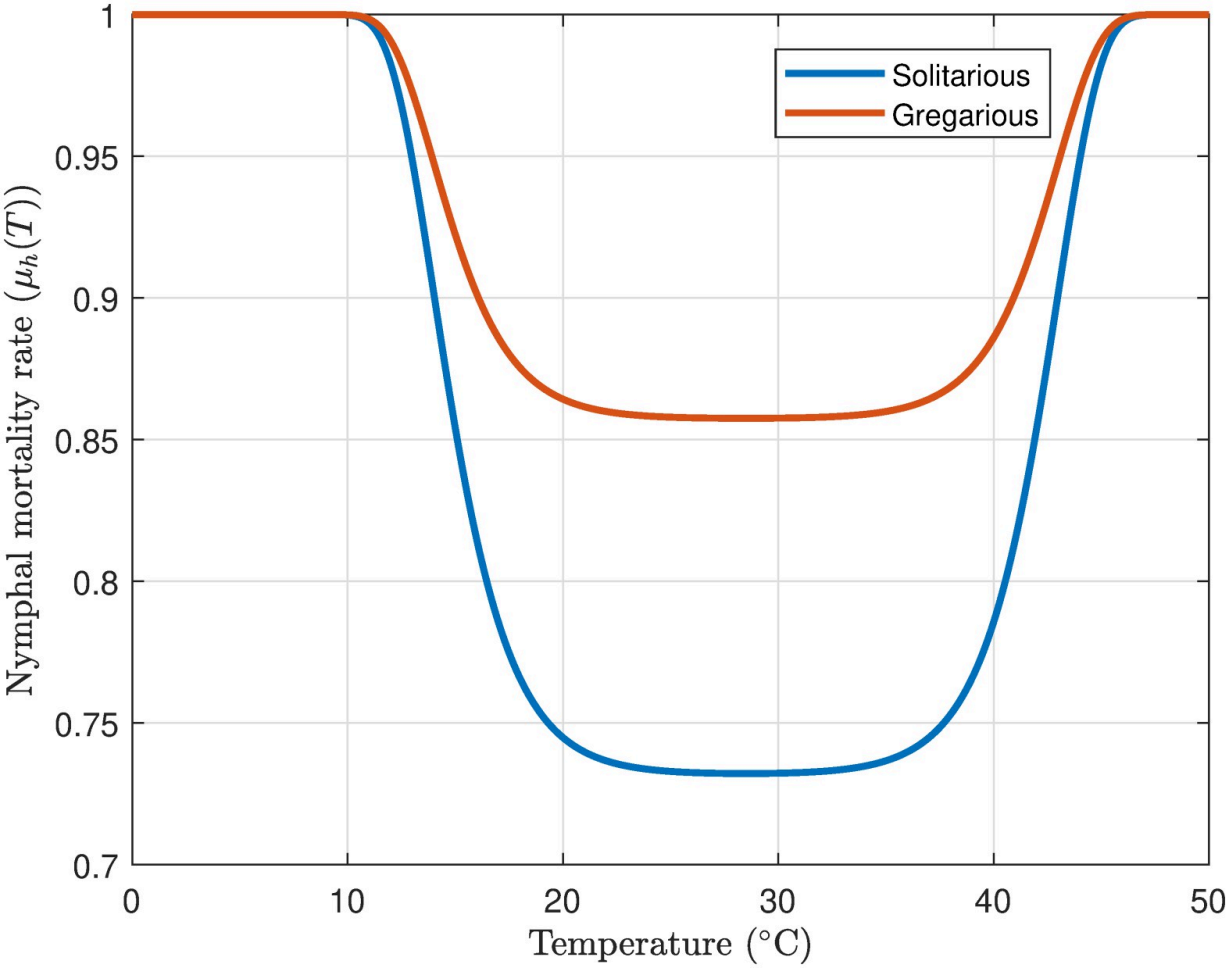
Profile of solitarious and gregarious nymphal mortality rate as a function of air temperature.

Fig 6 illustrates the simulated effect of temperature on hopper survival. The model anticipates a non-linear association between temperature and hopper mortality, with survival rates decreasing markedly as temperatures stray from the optimal range. Parameter values *M* = 0.1525°C for the solitarious phase and *M* = 0.282°C for the gregarious phase, *Q* = 2°*C*, *T*_max_ = 35°C, and *T*_min_ = 15°C are utilized. These findings are consistent with ecological observations suggesting that extreme temperatures can exert physiological stress on hoppers, increasing their vulnerability to diseases, predation, and developmental anomalies, thereby ultimately leading to elevated mortality rates.

#### 3.1.6 Adult mortality

Adult desert locusts demonstrate a high degree of susceptibility to environmental conditions, with temperature being a particularly critical factor. The temperature-dependent mortality rate is denoted by (*μ*_*s*_(*T*), *μ*_*g*_(*T*)). Temperature also exerts a significant influence on their migratory behavior. A temperature range of 20–22°C is requisite for flight, while 22–24°C is conducive to migration. Importantly, temperatures exceeding 27°C prompt mass take-off. Conversely, when temperatures surpass 40°C, their activity begins to diminish [55, 56].

**Fig 7:**
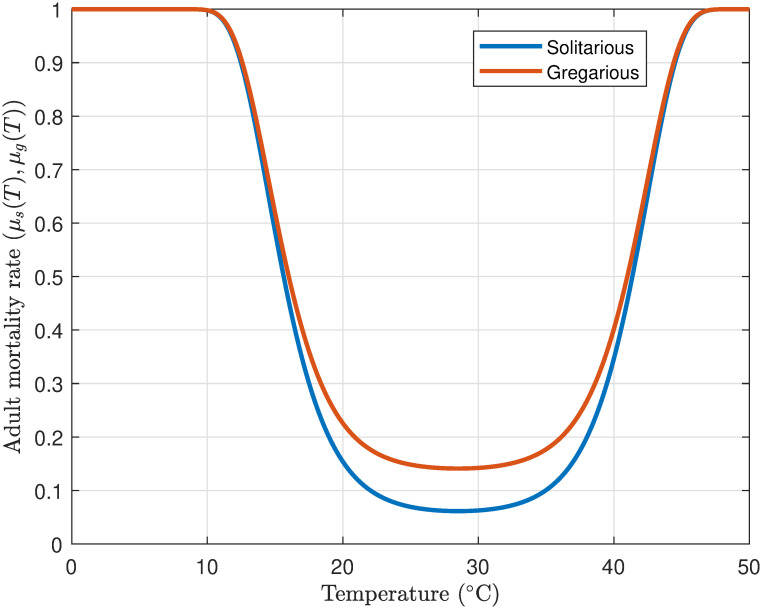
Profile of solitarious and gregarious temperature dependent adult mortality.

Fig 7 illustrates the temperature-dependent mortality rates of adult solitarious (*μ*_*s*_(*T*)) and gregarious (*μ*_*g*_(*T*)) desert locusts. The U-shaped curves demonstrate that mortality rates are minimized within the optimal temperature range of 20–40°C, wherein locusts benefit from enhanced food access and reduced physiological stress. Beyond this range, mortality escalates markedly as extreme temperatures restrict food availability and heighten stress, particularly when locusts are not engaged in flight or migration. Gregarious locusts persistently exhibit elevated mortality rates in comparison to solitarious locusts, likely attributable to increased competition for resources within swarms. This underscores the profound impact of temperature and resource availability on the survival dynamics of locusts.

### 3.2 Basic properties of the model

We analyze the fundamental dynamical properties of the non-autonomous system (4). Let *μ* represent the minimum mortality rate among all life stages (*μ*_*e*_, *μ*_*h*_, *μ*_*b*_, *μ*_*s*_, *μ*_*g*_). The right-hand side of the model (4) consists of smooth functions in terms of the variables *E*, *H*, *B*, *S*, and *G*, with all parameters being non-negative. We can express the system as follows:

$$\begin{array}{c} x^{\prime }=F\left(x\right), \end{array}$$ (10)

where *x* = (*E*, *H*, *B*, *S*, *G*)^*T*^ and $ℱ:[0,\infty )\to {\mathbb{R}}^{5}$. Defining the total locust population at time *t* as *N*(*t*) = *E*(*t*) + *H*(*t*) + *B*(*t*) + *S*(*t*) + *G*(*t*).

$$\begin{array}{c} \frac{dN}{dt}\leq \left(1-\eta \right)\phi \left(t\right)(1-\frac{E(t)}{K})\left(S\left(t\right)+\psi G\left(t\right)\right)-\mu N\left(t\right),\;t>0. \end{array}$$ (11)

To investigate the long-term behavior of the locust population under fluctuating temperature conditions, we hypothesize that the population converges to a periodic steady state. Building upon the work of [57, 58], we assume that for the *ω*-periodic function $\phi (T)\in C^{1}(0,{\mathbb{R}}_{+})$, there exists a positive constant *h*_0_ such that:

$$\begin{array}{c} \left(1-\eta \right)\phi \left(t\right)(1-\frac{E(t)}{K})\left(S\left(t\right)+\psi G\left(t\right)\right)-\mu \left(t\right)N<0,\;\forall N\geq h_{0}. \end{array}$$ (12)

**Lemma 3.1**. *For any* $x\in \Gamma =C([0,{\mathbb{R}}_{+}^{5}])$, *the model* (4) *admits a unique non-negative solution through x, and all solutions are ultimately and uniformly bounded*.

*Proof.* Let *x*(*t*) = (*E*(*t*), *H*(*t*), *B*(*t*), *S*(*t*), *G*(*t*))^*T*^ be a vector-valued function representing the locust population at time *t*, with initial condition *x*(0) = (*E*(0), *H*(0), *B*(0), *S*(0), *G*(0)). We can rewrite the system (4) as:

$$\begin{array}{c} \frac{dx}{dt}=f\left(t,x\left(t\right)\right),\;t\geq 0,\;x\left(0\right)=x_{0}, \end{array}$$

where

$$\begin{array}{c} f\left(t,x\left(t\right)\right)=[\begin{array}{c} \left(1-\eta \right)\phi \left(t\right)(1-\frac{x_{1}\left(0\right)}{K})\left(x_{4}\left(0\right)+\psi x_{5}\left(0\right)\right)-(\sigma \left(t\right)+\mu_{e}\left(t\right))x_{1}\left(0\right)\\ \theta \sigma \left(t\right)x_{1}\left(0\right)-(\gamma_{h}\left(t\right)+\mu_{h}\left(t\right))x_{2}\left(0\right)\\ \left(1-\theta \right)\sigma \left(t\right)x_{1}\left(0\right)-(\gamma_{b}\left(t\right)+\mu_{b}\left(t\right))x_{3}\left(0\right)\\ \gamma_{h}\left(t\right)x_{2}\left(0\right)+\beta_{2}x_{5}\left(0\right)-\left(\beta_{1}+\mu_{s}\left(t\right)\right)x_{4}\left(0\right)\\ \gamma_{b}\left(t\right)x_{3}\left(0\right)+\beta_{1}x_{4}\left(0\right)-\left(\beta_{2}+\mu_{g}\left(t\right)\right)x_{5}\left(0\right) \end{array}]. \end{array}$$

For any $x\in \Gamma =C([0,\infty ),{\mathbb{R}}_{+}^{5})$, the function *f*(*t*, *x*(*t*)) is continuous and Lipschitz continuous with respect to *x* on compact subsets of ℝ×Γ [57]. Therefore, there exists a unique solution to system (4) for any initial condition *x*(0) ∈ Γ. Moreover, since *f*_*i*_(*t*, *ν*) ≥ 0 whenever *ν* ≥ 0 and *ν*_*i*_(0) = 0, the non-negative orthant Γ is positively invariant for the system (4) [59].

Considering the total locust population *N*(*t*), its derivative satisfies Eq (13). By applying the comparison principle [60], we conclude that the solutions of the system (4) exist for all *t* ≥ 0. Furthermore,

$$\begin{array}{c} \underset{t\to \infty }{\text{lim}\;\text{sup}}\left(E\left(t\right)+H\left(t\right)+B\left(t\right)+S\left(t\right)+G\left(t\right)\right)\leq N^{*}\left(t\right), \end{array}$$

where N*(*t*) is the unique periodic solution of

$$\begin{array}{c} \frac{dN^{*}}{dt}=\left(1-\eta \right)\phi \left(t\right)(1-\frac{E(t)}{K})\left(S\left(t\right)+\psi G\left(t\right)\right)-\mu \left(t\right)N^{*},\;t>0. \end{array}$$ (13)

given by:

$$\begin{array}{cc} N^{*}\left(t\right)= & e^{-\int_{0}^{t}\mu \left(\tau \right)d\tau }\times [\int_{0}^{t}\left(1-\eta \right)\phi \left(\tau \right)(1-\frac{E(\tau )}{K})\left(S\left(\tau \right)+\psi G\left(\tau \right)\right)\;\text{exp}\;(\int_{0}^{s}\mu \left(\xi \right)d\xi )d\tau \\ & +\frac{\int_{0}^{\omega }\left(1-\eta \right)\phi \left(\tau \right)(1-\frac{E(\tau )}{K})\left(S\left(\tau \right)+\psi G\left(\tau \right)\right)\;\text{exp}\;(\int_{0}^{u}\mu \left(\zeta \right)d\zeta )d\tau }{e^{\int_{0}^{\omega }\mu \left(\zeta \right)d\zeta }-1}]. \end{array}$$

Hence, all solutions of the model (4) are ultimately bounded. Additionally, from Eq (13), we have N* < 0 whenever N* > *h*_0_, implying that all solutions are uniformly bounded [57, 58].

### 3.3 Theoretical analysis of the autonomous model

In this section, we examine the dynamics of the autonomous version of model (4). This simplification assumes that model parameters are constant, and independent of temperature. Consequently, we set *ϕ*(*T*) = *ϕ*, *σ*(*T*) = *σ*, *γ*(*T*) = *γ*, *μ*_*e*_(*T*) = *μ*_*e*_, *μ*_*h*_(*T*) = *μ*_*h*_, and *μ*_*b*_(*T*) = *μ*_*b*_. Under these conditions, system (4) reduces to:

$$\begin{array}{cc} \frac{dE}{dt} & =\left(1-\eta \right)\phi (1-\frac{E}{K})(S+\psi G)-(\sigma +\mu_{e})E,\\ \frac{dH}{dt} & =\theta \sigma E-(\gamma_{h}+\mu_{h})H,\\ \frac{dB}{dt} & =\left(1-\theta \right)\sigma E-(\gamma_{b}+\mu_{b})B,\\ \frac{dS}{dt} & =\gamma_{h}H+\beta_{2}G-\left(\beta_{1}+\mu_{s}\right)S,\\ \frac{dG}{dt} & =\gamma_{b}B+\beta_{1}S-\left(\beta_{2}+\mu_{g}\right)G. \end{array}$$ (14)

### 3.4 Basic properties of model

**Theorem 3.1**. *For any initial condition within the non-negative* Γ, *the autonomous model* (14) *possesses a unique, globally defined, bounded solution that remains within* Γ.

*Proof*. Given the smoothness of ℱ within Γ, the Cauchy problem associated with Eq (14) admits a unique maximal solution for any initial condition in Γ. To demonstrate the positivity of solutions, we observe that the vector field defined by ℱ is either tangent to or points inwards on the boundary of Γ:

$$\begin{array}{cc} \frac{dE}{dt}{|}_{E=0} & =(1-\eta )\phi (S+\psi G)\geq 0,\;\forall S,G\geq 0,\\ \frac{dH}{dt}{|}_{H=0} & =\theta \sigma E\geq 0,\;\forall E\geq 0,\\ \frac{dB}{dt}{|}_{B=0} & =(1-\theta )\sigma E\geq 0,\;\forall E\geq 0,\\ \frac{dS}{dt}{|}_{S=0} & =\gamma_{h}H+\beta_{2}G\geq 0,\;\forall H,G\geq 0,\\ \frac{dG}{dt}{|}_{G=0} & =\gamma_{b}B+\beta_{1}S\geq 0,\;\forall B,S\geq 0. \end{array}$$

Consequently, solutions with non-negative initial conditions remain non-negative. Moreover, as the system is locally Lipschitz continuous, standard existence theorems for ordinary differential equations guarantee the existence of solutions for all *t* ≥ 0.

To establish boundedness, consider the total population *N*(*t*) = *E*(*t*) + *H*(*t*) + *B*(*t*) + *S*(*t*) + *G*(*t*). Its derivative satisfies:

$$\begin{array}{cc} \frac{dN}{dt} & =\left(1-\eta \right)\phi \left(S+\psi G\right)-\left(1-\eta \right)\phi \frac{E(S+\psi G)}{K}-\mu_{e}E-\mu_{h}H-\mu_{b}B-\mu_{s}S-\mu_{g}G\\ & \leq (1-\eta )\phi 2K-\mu N(t), \end{array}$$

where *μ* = min{*μ*_*e*_, *μ*_*h*_, *μ*_*b*_, *μ*_*s*_, *μ*_*g*_} and we have used the fact that *S*, *G* ≤ *K* and 1 + *ψ* < 2. Applying Gronwall’s inequality, we obtain:

$$0\leq N\left(t\right)\leq N\left(0\right)e^{-\mu t}+\frac{(1-\eta )\phi 2K}{\mu }.$$

As *t* → ∞, we have:

$$0\leq N\left(t\right)\leq \frac{(1-\eta )\phi 2K}{\mu }.$$

Hence, *N*(*t*) is bounded, implying that all components of the solution are also bounded within Γ.

#### 3.4.1 Stability of trivial equilibrium

This subsection explores the existence and stability of equilibrium points for the autonomous model (14). The autonomous model (14) admits a trivial equilibrium point, denoted by *P*_0_ = (0, 0, 0, 0, 0). To investigate the stability of *P*_0_, we employ the next-generation matrix approach [61, 62].

For the system (14), we define:

-
$R_{i}(x)$ : the rate of recruitment into compartment *i*,
-
$V_{i}^{+}(x)$ : the rate of transfer into compartment *i*,
-
$V_{i}^{-}(x)$ : the rate of transfer out of compartment *i*.

Consequently, the system can be expressed as:

$$\begin{array}{c} x_{i}^{\prime }=R_{i}\left(x\right)-V_{i}\left(x\right), \end{array}$$

where $V_{i}=V_{i}^{-}(x)-V_{i}^{+}(x)$ and *i* = 1, …, 5.

For the specific system (14), we have:

$$\begin{array}{c} R\left(x\right)=(\begin{array}{c} \left(1-\eta \right)\phi (1-\frac{E}{K})\left(S+\psi G\right)\\ 0\\ 0\\ 0\\ 0 \end{array}) \end{array}$$

and

$$\begin{array}{c} V\left(x\right)=(\begin{array}{c} c_{1}E\\ c_{2}H-\theta \sigma E\\ c_{3}B-\left(1-\theta \right)\sigma E\\ c_{4}S-\gamma_{h}H-\beta_{2}G\\ c_{5}G-\gamma_{b}B-\beta_{1}S \end{array}), \end{array}$$

where *c*_1_ = *σ* + *μ*_*e*_, *c*_2_ = *γ*_*h*_ + *μ*_*h*_, *c*_3_ = *γ*_*b*_ + *μ*_*b*_, *c*_4_ = *β*_1_ + *μ*_*s*_, and *c*_5_ = *β*_2_ + *μ*_*g*_.

The next-generation matrix, *RV*^−1^, is obtained from the Jacobian matrices of R and V evaluated at the equilibrium point *P*_0_, denoted by *R* and *V*, respectively. In this case:

$$\begin{array}{c} R=(\begin{array}{ccccc} 0 & 0 & 0 & (1-\eta )\phi & (1-\eta )\phi \psi \\ 0 & 0 & 0 & 0 & 0\\ 0 & 0 & 0 & 0 & 0\\ 0 & 0 & 0 & 0 & 0\\ 0 & 0 & 0 & 0 & 0 \end{array}) \end{array}$$

and

$$\begin{array}{c} V=(\begin{array}{ccccc} c_{1} & 0 & 0 & 0 & 0\\ -\theta \sigma & c_{2} & 0 & 0 & 0\\ -(1-\theta )\sigma & 0 & c_{3} & 0 & 0\\ 0 & -\gamma_{h} & 0 & c_{4} & -\beta_{2}\\ 0 & 0 & -\gamma_{b} & -\beta_{1} & c_{5} \end{array}). \end{array}$$

The basic offspring number, $N_{0}$, represents the expected number of female offspring produced by a single female over her lifetime. It is calculated as the spectral radius of the next-generation matrix *RV*^−1^. The number $N_{0}$ can be decomposed into two components: the average number of eggs laid by a solitarious adult female denoted by $N_{0S}$ and the number of eggs laid by a gregarious adult female represent by $N_{0G}$.

Therefore,

$$\begin{array}{c} N_{0}=N_{0S}+N_{0G}, \end{array}$$ (15)

The specific formulas for $N_{0S}$ and $N_{0G}$ are as follows:

$$\begin{array}{cc} N_{0S} & =\frac{\left(1-\eta \right)\phi (\theta \sigma \gamma_{h}c_{3}c_{5}+\left(1-\theta \right)\sigma \gamma_{b}\beta_{2}c_{2})}{c_{1}c_{2}c_{3}(c_{4}c_{5}-\beta_{1}\beta_{2})},\\ N_{0G} & =\frac{\left(1-\eta \right)\psi \phi (\left(1-\theta \right)\sigma \gamma_{b}c_{2}c_{4}+\theta \sigma \gamma_{h}\beta_{1}c_{3})}{c_{1}c_{2}c_{3}(c_{4}c_{5}-\beta_{1}\beta_{2})}. \end{array}$$

Based on Theorem 2 from [61], we have:

**Theorem 3.2**. *The trivial equilibrium P*_0_ *is locally asymptotically stable (LAS) if* $N_{0}<1$ *and unstable if* $N_{0}>1$.

#### 3.4.2 Existence and stability of non-trivial equilibrium

When $N_{0}>1$, the system (14) possesses a non-trivial equilibrium P** = (E**, H**, B**, S**, G**) given by:

$$\begin{array}{cc} E^{**} & =\frac{K}{N_{0}}\left(N_{0}-1\right),\;H^{**}=\frac{\theta K\sigma }{c_{2}N_{0}}\left(N_{0}-1\right),\;B^{**}=\frac{(1-\theta )K\sigma }{c_{3}N_{0}}\left(N_{0}-1\right),\\ S^{**} & =\frac{\sigma K(c_{2}\gamma_{b}\beta_{2}\left(1-\theta \right)+\theta \gamma_{h}c_{3}c_{4})}{c_{2}c_{3}N_{0}}\left(N_{0}-1\right),\;G^{**}=\frac{\sigma K(c_{2}c_{4}\left(1-\theta \right)\gamma_{b}+\theta \gamma_{h}c_{3}\beta_{1})}{c_{2}c_{3}N_{0}}\left(N_{0}-1\right). \end{array}$$

**Theorem 3.3**. *The non-trivial equilibrium* (P**) *is locally-asymptotically stable (LAS) when* $N_{0}>1$.

*Proof*. To establish the local asymptotic stability of P**, we analyze the Jacobian matrix of the autonomous model evaluated at P**, denoted by *J*(P**):

$$\begin{array}{c} J\left(P^{**}\right)=(\begin{array}{ccccc} -c_{1}-A_{1} & 0 & 0 & \frac{(1-\eta )\phi }{N_{0}} & \frac{(1-\eta )\phi \psi }{N_{0}}\\ \theta \sigma & -c_{2} & 0 & 0 & 0\\ (1-\theta )\sigma & 0 & -c_{3} & 0 & 0\\ 0 & \gamma_{h} & 0 & -c_{4} & \beta_{2}\\ 0 & 0 & \gamma_{b} & \beta_{1} & -c_{5} \end{array}), \end{array}$$ (16)

where

$$A_{1}=\frac{\left(1-\eta \right)\phi \sigma \left(N_{0}-1\right)\left(c_{2}\left(1-\theta \right)\gamma_{b}\left(\beta_{2}+c_{4}\psi \right)+c_{3}\theta \gamma_{h}\left(\beta_{1}\psi +c_{5}\right)\right)}{c_{2}c_{3}N_{0}}.$$

The matrix *J*(P**) can be decomposed into *J*(P**) = *D*+ *A*, where *D* is a diagonal matrix containing the diagonal elements of *J*(P**) and *A* is a matrix with the off-diagonal elements of *J*(P**). Thus,

$$D=(\begin{array}{ccccc} -c_{1}-A_{1} & 0 & 0 & 0 & 0\\ 0 & -c_{2} & 0 & 0 & 0\\ 0 & 0 & -c_{3} & 0 & 0\\ 0 & 0 & 0 & -c_{4} & 0\\ 0 & 0 & 0 & 0 & -c_{5} \end{array}),\;A=(\begin{array}{ccccc} 0 & 0 & 0 & \frac{(1-\eta )\phi }{N_{0}} & \frac{(1-\eta )\phi \psi }{N_{0}}\\ \theta \sigma & 0 & 0 & 0 & 0\\ (1-\theta )\sigma & 0 & 0 & 0 & 0\\ 0 & \gamma_{h} & 0 & 0 & \beta_{2}\\ 0 & 0 & \gamma_{b} & \beta_{1} & 0 \end{array}).$$

Since all off-diagonal elements of *J*(P**) are non-negative, *A* is a Metzler matrix. Furthermore, a straightforward calculation shows that *ρ*(−*D*^−1^*A*) < 1 when $N_{0}>1$, implying that *J*(P**) is also a stable Metzler matrix under this condition. Consequently, P** is locally asymptotically stable.

#### 3.4.3 Non-existence of backward bifurcation

To investigate the possibility of a backward bifurcation in our model, we use the Centre Manifold Theory approach outlined in [61, 63]. We analyze the autonomous version of model (14) for this study.

**Theorem 3.4**. *[Castillo-Chavez and Song* [63]*] Consider the following ordinary differential equations, with a parameter* λ:

$$\begin{array}{c} \frac{dx}{dt}=f\left(x,\lambda \right),\;f:R^{n}\times R\to R^{n}\;\;\text{and}\;\;f\in C^{2}\left(R^{n}\times R\right). \end{array}$$ (17)

*Without loss of generality, it is assumed that* **0** *is an equilibrium for system* (17) *for all values of the parameter* λ, *that is f*(0, λ) ≡ 0 *for all* λ. *Assume*:

*A1*: $A=D_{x}f(0,0)=\left(\frac{\partial f_{i}}{\partial x_{j}}(0,0)\right)$ *is the linearization matrix of system* (17) *around the equilibrium* **0** *with* λ *evaluated at* **0**. *Zero is a simple eigenvalue of A and all other eigenvalues of A have negative real parts*;
*A2: Matrix A has a nonnegative right eigenvector* **u** *and a left eigenvector* **v** *corresponding to the zero eigenvalue*.

*Let f*_*k*_ *be the kth component of f and*

$$\begin{array}{cc} a= & \sum_{k,i,j=1}^{n}v_{k}u_{i}u_{j}\frac{\partial^{2}f_{k}}{\partial x_{i}\partial x_{j}}\left(0,0\right),\\ b= & \sum_{k,i=1}^{n}v_{k}u_{i}\frac{\partial^{2}f_{k}}{\partial x_{i}\partial \lambda }\left(0,0\right). \end{array}$$

*The local dynamics of* (17) *around* **0** *are totally determined by a and b*.

- *a* > 0, *b* > 0. *When* λ < 0 *with* |λ| ≪ 1, **0** *is locally asymptotically stable, and there exists a positive unstable equilibrium; when* 0 < λ ≪ 1, **0** *is unstable and there exists a negative and locally asymptotically stable equilibrium*;
- *a* < 0, *b* < 0. *When* λ < 0 *with* |λ| ≪ 1, **0** *is unstable; when* 0 < λ ≪ 1, **0** *is locally asymptotically stable, and there exists a positive unstable equilibrium*;
- *a* > 0, *b* < 0. *When* λ < 0 *with* |λ| ≪ 1, **0** *is unstable, and there exists a locally asymptotically stable negative equilibrium; when* 0 < λ ≪ 1, **0** *is stable, and a positive unstable equilibrium appears*;
- *a* < 0, *b* > 0. *When* λ *changes from negative to positive*, **0** *changes its stability from stable to unstable. Correspondingly a negative unstable equilibrium becomes positive and locally asymptotically stable*.

Now, we will be use Theorem 3.4 to explore the possibility of backward bifurcation in (14) with egg-laying rates *ϕ*. To do so, a bifurcation parameter (ϕ*) is chosen, by solving for *ϕ* from $N_{0}=1$, giving

$$\begin{array}{c} \phi^{*}=\frac{c_{1}c_{2}c_{3}\left(\mu_{s}c_{5}+\beta_{1}\mu_{g}\right)}{\left(1-\eta \right)\sigma (\theta \gamma_{b}c_{3}\left(\beta_{1}\psi +c_{5}\right)+\left(1-\theta \right)\gamma_{h}c_{2}\left(\beta_{2}+c_{4}\psi \right))}. \end{array}$$ (18)

Let *J*_ϕ*_ denote the Jacobian of the system (14) evaluated at the extinction equilibrium point *P*^0^ and with *ϕ* = ϕ*. Thus,

$$\begin{array}{c} J_{\phi^{*}}=(\begin{array}{ccccc} -c_{1} & 0 & 0 & \left(1-\eta \right)\phi^{*} & \left(1-\eta \right)\psi \phi^{*}\\ \theta \sigma & -c_{2} & 0 & 0 & 0\\ (1-\theta )\sigma & 0 & -c_{3} & 0 & 0\\ 0 & \gamma_{h} & 0 & -c_{4} & \beta_{2}\\ 0 & 0 & \gamma_{b} & \beta_{1} & -c_{5} \end{array}) \end{array}$$ (19)

The Jacobian matrix *J*_ϕ*_ of the linearized system has a simple zero eigenvalue (with all other eigenvalues having negative real parts). Hence the theory based on Center Manifold Theory [63] can be used to analysed the dynamics of the system (14). We obtained the left eigenvector (*v*) corresponding to the zero eigenvalue given by *v* = (*v*_1_, *v*_2_, ⋯, *v*_5_)^*T*^, where

$$\begin{array}{cc} v_{1} & =(\frac{\beta_{2}\mu_{s}-\beta_{1}\mu_{g}}{c_{5}-\psi c_{4}})\frac{v_{5}}{\left(1-\eta \right)\phi^{*}},\;v_{2}=\frac{\gamma_{h}}{c_{2}}(\frac{\beta_{2}\mu_{s}-\beta_{1}\mu_{g}}{c_{5}-\psi c_{4}})v_{5},\\ v_{3} & =\frac{\gamma_{b}v_{5}}{c_{3}},\;v_{4}=(\frac{\beta_{2}\mu_{s}-\beta_{1}\mu_{g}}{c_{5}-\psi c_{4}})v_{5},\;v_{5}>0. \end{array}$$

The sign of *v*_1_ depends on the relationship between the numerator *β*_2_*μ*_*s*_ − *β*_1_*μ*_*g*_ and the denominator *c*_5_ − *ψc*_4_. Specifically, *v*_1_ < 0 when these terms have opposite signs, and *v*_1_ > 0 when they have the same sign.

The matrix *J*_ϕ*_ has a left eigenvector given by *u* = (*u*_1_, ⋯, *u*_5_), where

$$\begin{array}{cc} u_{1} & =\frac{(\beta_{2}\mu_{s}+\mu_{g}c_{4})c_{2}c_{3}u_{5}}{\sigma (\left(1-\theta \right)\gamma_{h}c_{2}c_{4}+\theta \gamma_{b}\beta_{1}c_{3})},\;u_{2}=\frac{(\beta_{2}\mu_{s}+\mu_{g}c_{4})\theta c_{3}u_{5}}{\left(\left(1-\theta \right)\gamma_{h}c_{2}c_{4}+\theta \gamma_{b}\beta_{1}c_{3}\right)}\\ u_{3} & =\frac{(\beta_{2}\mu_{s}+\mu_{g}c_{4})\left(1-\theta \right)c_{2}u_{5}}{\left(\left(1-\theta \right)\gamma_{h}c_{2}c_{4}+\theta \gamma_{b}\beta_{1}c_{3}\right)},\;u_{4}=\frac{(\beta_{2}\mu_{s}+\mu_{g}c_{4})\theta c_{3}u_{5}}{c_{4}(\left(1-\theta \right)c_{2}c_{4}+\theta \beta_{1}c_{3})}+\frac{\beta_{2}u_{5}}{c_{4}},\;u_{5}>0 \end{array}$$

It follows then, after some algebraic manipulations, that

$$\begin{array}{c} a=\frac{-2(1-\eta )\phi }{K}v_{1}u_{1}\left(u_{4}+\psi u_{5}\right)\\ b=\left(1-\eta \right)v_{1}\left(u_{4}+\psi u_{5}\right) \end{array}$$

Note that the coefficients *a* and *b* have opposite signs determined by the sign of *v*_1_. Thus, applying Theorem 3.4, we conclude that:

**Theorem 3.5**. *The model* (14) *exhibits a forward bifurcation at* $N_{0}=1$ *whenever v*_1_ > 0.

**Theorem 3.6**. *The extinction equilibrium (P*_0_*) is globally-asymptotically stable (GAS) when* $N_{0}\leq 1$ *and unstable otherwise*.

*Proof*. Rewriting the system, we have:

$$\begin{array}{c} \frac{dx}{dt}=(\begin{array}{c} \frac{dE}{dt}\\ \frac{dH}{dt}\\ \frac{dB}{dt}\\ \frac{dS}{dt}\\ \frac{dG}{dt} \end{array})=(\begin{array}{c} \phi \left(1-\eta \right)(1-\frac{E}{K})(S+\psi G)-\left(\sigma +\mu_{e}\right)E\\ \theta \sigma E-(\gamma_{h}+\mu_{h})\\ \left(1-\theta \right)\sigma E-\left(\gamma_{b}+\mu_{b}\right)B\\ \gamma_{h}H+\beta_{2}G-\left(\beta_{1}+\mu_{s}\right)S\\ \gamma_{b}B+\beta_{1}S-\left(\beta_{2}+\mu_{g}\right)G \end{array}). \end{array}$$ (20)

To rewrite this system in the desired form $\frac{dx}{dt}=(R-V)x-Mx$, we first isolate the linear and nonlinear terms. Linear terms:

$$\begin{array}{cc} R & =(\begin{array}{ccccc} 0 & 0 & 0 & 0 & 0\\ \theta \sigma & 0 & 0 & 0 & 0\\ (1-\theta )\sigma & 0 & 0 & 0 & 0\\ 0 & \gamma_{h} & 0 & 0 & \beta_{2}\\ 0 & 0 & \gamma_{b} & \beta_{1} & 0 \end{array}),\\ V & =(\begin{array}{ccccc} \sigma +\mu_{e} & 0 & 0 & 0 & 0\\ 0 & \gamma_{h}+\mu_{h} & 0 & 0 & 0\\ 0 & 0 & \gamma_{b}+\mu_{b} & 0 & 0\\ 0 & 0 & 0 & \beta_{1}+\mu_{s} & 0\\ 0 & 0 & 0 & 0 & \beta_{2}+\mu_{g} \end{array}). \end{array}$$ (21)

Nonlinear terms (which will form *Mx*):

$$\begin{array}{c} Mx=(\begin{array}{c} \phi \left(1-\eta \right)\frac{E}{K}\left(S+\psi G\right)\\ 0\\ 0\\ 0\\ 0 \end{array}). \end{array}$$ (22)

Thus, the system can be written as $\frac{dX}{dt}=(R-V)x-Mx$. Since *Mx* is non-negative for *x* ≥ 0, we can use the comparison theorem [59, 60]. Define $\hat{X}$ as the solution to the system: $\frac{d\hat{x}}{dt}=(R-V)\hat{x}.$ Because $\frac{dx}{dt}=(R-V)x-Mx\leq (R-V)x$, we have $\frac{dx}{dt}\leq \frac{d\hat{x}}{dt}$.

Now we analyze the stability of the system $\frac{d\hat{x}}{dt}=(R-V)\hat{x}$. The matrix *R* − *V* is:

$$\begin{array}{c} R-V=(\begin{array}{ccccc} -(\sigma +\mu_{e}) & 0 & 0 & 0 & 0\\ \theta \sigma & -(\gamma_{h}+\mu_{h}) & 0 & 0 & 0\\ (1-\theta )\sigma & 0 & -(\gamma_{b}+\mu_{b}) & 0 & 0\\ 0 & \gamma_{h} & 0 & -(\beta_{1}+\mu_{s}) & \beta_{2}\\ 0 & 0 & \gamma_{b} & \beta_{1} & -(\beta_{2}+\mu_{g}) \end{array}). \end{array}$$ (23)

Since all the diagonal elements of *R* − *V* are negative and all off-diagonal elements are non-positive, the matrix *R* − *V* is Hurwitz. This implies that the system $\frac{d\hat{x}}{dt}=(R-V)\hat{x}$ is globally asymptotically stable at the origin. Therefore, by the comparison theorem [59, 60], the original system $\frac{dx}{dt}\leq (R-V)x$ is also globally asymptotically stable at the trivial equilibrium point *P*_0_ = (0, 0, 0, 0, 0).

### 3.5 Theoretical analysis of non-autonomous model

In this section, we explore the dynamical properties of the non-autonomous model (4). The non-autonomous model (4) has a unique trivial equilibrium point, denoted by $P_{0}^{*}=(0,0,0,0,0,0)$, and possibly positive periodic solution(s), denoted by $P_{1}^{**}=(E^{**}(t),H^{**}(t),B^{**}(t),S^{**}(t),G^{**}(t))$, which satisfies the following periodic system:

$$\begin{array}{cc} \frac{dE^{*}\left(t\right)}{dt} & =\left(1-\eta \right)\phi \left(T\right)(1-\frac{E^{*}\left(t\right)}{K})(S^{*}\left(t\right)+\psi G^{*}\left(t\right))-(\sigma \left(T\right)+\mu_{e}\left(T\right))E^{*}\left(t\right),\\ \frac{dH^{*}\left(t\right)}{dt} & =\theta \sigma \left(T\right)E^{*}\left(t\right)-(\gamma_{h}\left(T\right)+\mu_{h}\left(T\right))H^{*}\left(t\right),\\ \frac{dB^{*}\left(t\right)}{dt} & =\left(1-\theta \right)\sigma \left(T\right)E^{*}\left(t\right)-(\gamma_{b}\left(T\right)+\mu_{b}\left(T\right))B^{*}\left(t\right),\\ \frac{dS^{*}\left(t\right)}{dt} & =\gamma_{h}\left(T\right)H^{*}\left(t\right)+\beta_{2}G^{*}\left(t\right)-\left(\beta_{1}+\mu_{s}\right)S^{*}\left(t\right),\\ \frac{dG^{*}\left(t\right)}{dt} & =\gamma_{b}\left(T\right)B^{*}\left(t\right)+\beta_{1}S^{*}\left(t\right)-\left(\beta_{2}+\mu_{g}\right)G^{*}\left(t\right). \end{array}$$ (24)

#### 3.5.1 Computation of vectorial offspring ratio

The local-asymptotic stability of the positive periodic trivial equilibrium($P_{0}^{*}$) can be established using a threshold parameter called the basic offspring ratio [64]. We computed the basic reproduction ratio for the model (4) using the theory developed in [64] and using the approach in [39, 57, 58, 65, 66]. The next generation matrix *F*(*t*) (of the new eggs deposited) and the M-Matrix *V*(*t*) (of the remaining transfer terms), associated with the non-autonomous model (14) (linearized at the trivial equilibrium $P_{0}^{*}$), are given, respectively, by

$$\begin{array}{c} F\left(t\right)=(\begin{array}{ccccc} 0 & 0 & 0 & \left(1-\eta )\phi (T\right) & \psi (1-\eta )\phi (T)\\ 0 & 0 & 0 & 0 & 0\\ 0 & 0 & 0 & 0 & 0\\ 0 & 0 & 0 & 0 & 0\\ 0 & 0 & 0 & 0 & 0 \end{array}), \end{array}$$

$$\begin{array}{c} V\left(t\right)=(\begin{array}{ccccc} c_{1} & 0 & 0 & 0 & 0\\ -\theta \sigma (T) & c_{2} & 0 & 0 & 0\\ -(1-\theta )\sigma (T) & 0 & c_{3} & 0 & 0\\ 0 & -\gamma_{h}\left(T\right) & 0 & c_{4} & -\beta_{2}\\ 0 & 0 & -\gamma_{b}\left(T\right) & -\beta_{1} & c_{5} \end{array}). \end{array}$$

Noticed that *F*(*t*) is non-negative and −*V*(*t*) is cooperative. Let *x*(*t*) = (*E*(*t*), *H*(*t*), *B*(*t*), *S*(*t*), *G*(*t*))^*T*^, then the linearization of (24) can be re-written in the form

$$\begin{array}{c} \frac{dx(t)}{dt}=(F(t)-V(t))x\left(t\right) \end{array}$$

Following the approach of [57, 64], let *Y*(*t*, *s*), *t* ≥ *s* is the evolution operator of the linear *ω*−periodic system $\frac{dy}{dt}=-V(t)y(t),\;t\geq s$, that is for each s∈ℝ, the 5 × 5 matrix *Y*(*t*, *s*) satisfies

$$\begin{array}{c} \frac{d}{dt}Y\left(t,s\right)=-V\left(t\right)Y\left(t,s\right),\;Y\left(s,s\right)=I,\;t\geq s, \end{array}$$

where *I* is the 5 × 5 identity matrix. Let ${\mathbb{C}}_{\omega }$ be the Banach space of all *ω*−periodic functions equipped with the maximum norm. Suppose $\alpha (s)\in {\mathbb{C}}_{\omega }$ is the initial distribution of new eggs, then the rate at which new eggs in the breeding habitat at time *s* are generating (hatching) is given by *F*(*s*)*α*(*s*) [58, 64]. Likewise, the distribution of new eggs time *s* and remaining in adults at a later time *t* is *Y*(*t*, *s*)*F*(*s*)*α*(*s*). Therefore

$$\begin{array}{c} \psi \left(t\right)=\int_{-\infty }^{t}Y\left(t,s\right)F\left(s\right)\alpha \left(s\right)ds=\int_{0}^{\infty }Y\left(t,t-a\right)F\left(t-a\right)\alpha \left(s\right)\left(t-a\right)da \end{array}$$ (25)

gives the cumulative distribution of new eggs at time *t* that are produced by all female adult desert locusts (*α*(*s*)) introduced at sometimes before *s* = *t*. Define the linear operator $L:{\mathbb{C}}_{\omega }\to {\mathbb{C}}_{\omega }$ by

$$\begin{array}{c} \left(L\alpha \right)\left(t\right)=\int_{0}^{\infty }Y\left(t,t-a\right)F\left(t-a\right)\alpha \left(t-a\right)da\;\forall t\in R,\;\alpha \in C_{\omega }. \end{array}$$ (26)

Suppose *ρ*(*L*) is the spectral radius of *L*, then the basic offspring ratio $\left(N_{0\omega }\right)$ is given by *ρ*(*L*) [64]. It is easy to show that, in addition to Assumptions *A*_1_ to *A*_5_ of [64] satisfied by the autonomous system, the non-autonomous model (4) can be shown to satisfy the additional Assumptions *A*_6_ and *A*_7_ of [64]. Thus, the result below follows from Theorem 2.2 in [64].

**Theorem 3.7**. *The trivial equilibrium*, $P_{0}^{*}$, *of the non-autonomous model* (4), *is LAS in* $C([0],{\mathbb{R}}_{+}^{5})$ *if* $N_{0\omega }<1$, *and unstable if* $N_{0\omega }>1$.

### 3.6 Sensitivity analysis

To evaluate the relative influence of model parameters on locust population growth, we conducted a sensitivity analysis using the Partial Rank Correlation Coefficient (PRCC). This method quantifies the monotonic relationship between each parameter and the basic offspring number ($N_{0}$), accounting for potential interactions among parameters. The analysis was performed under both constant and fluctuating temperature conditions to assess the impact of temperature variability on parameter sensitivity.

**Fig 8:**
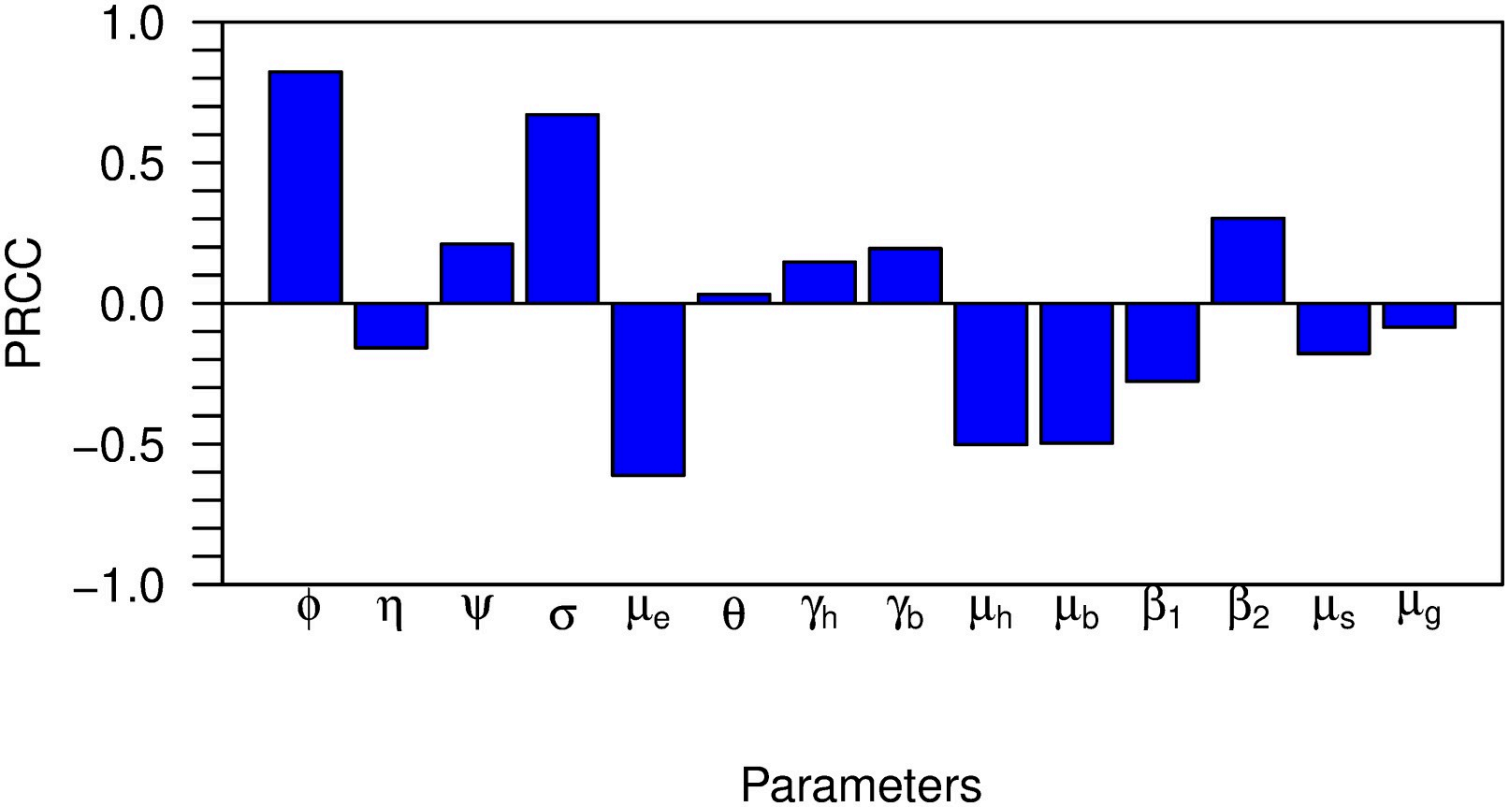
Partial rank correlation coefficients (PRCCs) between model parameters and the basic offspring number ($N_{0}$).

The parameters in the desert locust population dynamics model are essential in determining the fundamental offspring number, $N_{0}$, which impacts the potential for population expansion. Fig 8 demonstrates the correlation between model parameters and the offspring number. The parameter *ϕ* represents the daily fecundity rate of female locusts. Elevated values of *ϕ* enhance the egg-laying capacity, substantially contributing to population growth. The parameter *η* signifies the likelihood of males being encountered, which subsequently affects the probability of successful oviposition. When the density of adult males is elevated, the ratio of females declines, thereby reducing the number of offspring. Conversely, a diminished male ratio (higher (1 − *η*)) enhances reproductive potential, as a greater proportion of females are available for oviposition.

The modification parameter *ψ* addresses the diminished fecundity observed in gregarious locusts compared to solitarious ones. Given that gregarious females deposit fewer eggs, *ψ* diminishes reproductive output during gregarious phases. The egg-hatching rate *σ* regulates the pace at which eggs develop into hoppers. Elevated *σ* values expedite the transition from egg to hopper, thus enhancing the potential for population surges. Conversely, the egg mortality rate *μ*_*e*_ adversely affects population growth because increased mortality diminishes the quantity of viable eggs that can hatch.

The parameters *γ*_*h*_ and *γ*_*b*_ denote the developmental rates of solitarious and gregarious hoppers, respectively. Accelerated development (higher *γ*_*h*_ or *γ*_*b*_) shortens the hopper stage while increasing the probability of survival to adulthood. Nevertheless, elevated hopper mortality rates, *μ*_*h*_ for solitarious and *μ*_*b*_ for gregarious locusts, decrease population levels by reducing the number of hoppers reaching maturity. Survival rates during these phases are critical, as even minimal increases in mortality can severely constrain population growth.

The parameter *θ* governs the proportion of solitarious versus gregarious hatching. A higher *θ* indicates a propensity for more solitarious hatching, which typically exhibits higher fecundity. Parameters *β*_1_ and *β*_2_ pertain to the survival rates of adult locusts in solitarious and gregarious phases, respectively. Higher survival rates (especially *β*_2_ in gregarious adults) promote population sustainability by enabling a greater number of adults to reproduce.

Finally, *μ*_*s*_ and *μ*_*g*_ represent the mortality rates for solitarious and gregarious adults. Lower adult mortality extends the reproductive window, facilitating population growth. In aggregate, these parameters interact to define the overall dynamics of desert locust populations, where fecundity, survival, and mortality rates at each lifecycle stage are instrumental in forecasting and managing population outbreaks.

### 3.7 Numerical simulation

In this subsection, we examine the numerical results of the model. To analyze the dynamical behavior and evaluate the stability of the trivial equilibrium point, numerical simulations were performed utilizing the ODE45 solver in MATLAB. Baseline parameter values were sourced from Table 2.

**Table 2:** Description of parameters.

Parameters	Description	Range	Baseline Value	Source
*ϕ*	Eggs oviposition rate	0.933–4.44	3.556 day^−1^	[38]
*ψ*	Modification parameter	-	0.35 (dimensionless)	[37]
*η*	Adult male ratio	0.302–0.501	0.483 (dimensionless)	[40]
*K*	Carrying capacity of egg	16, 000–80, 000	80, 000 Locusts m^−2^	[9, 38]
*σ*	Eggs hatching rate	0.0154–0.1	0.0714 day^−1^	[42]
*μ*_*e*_	Egg mortality rate	0.05–0.65	0.33 day^−1^	[37, 43]
*θ*	Portion of solitarious hopper	0–1	0.65 (dimensionless)	Assume
*γ*_*h*_	Hopper development rate	0.0208–0.0333	0.0263 day^−1^	[42]
*γ*_*b*_	Band development rate	0.0175–0.04	0.0243 day^−1^	[42]
*μ*_*h*_	Mortality rate of hoppers	-	0.7332 day^−1^	[38]
*μ*_*b*_	Mortality rate of bands	-	0.8575 day^−1^	[38]
*μ*_*s*_	Mortality rate of solitarious adults	0.0067–0.0111	0.0083 day^−1^	[38]
*μ*_*g*_	Mortality rate of gregarious adults	0.0067–0.0111	0.0111 day^−1^	[38]
*β*_1_	Gregarisation rate	0.125–0.25	0.25 hr^−1^	[41, 44]
*β*_2_	The dissociation rate	0.125–0.25	0.25 hr^−1^	[41, 44]

**Fig 9:**
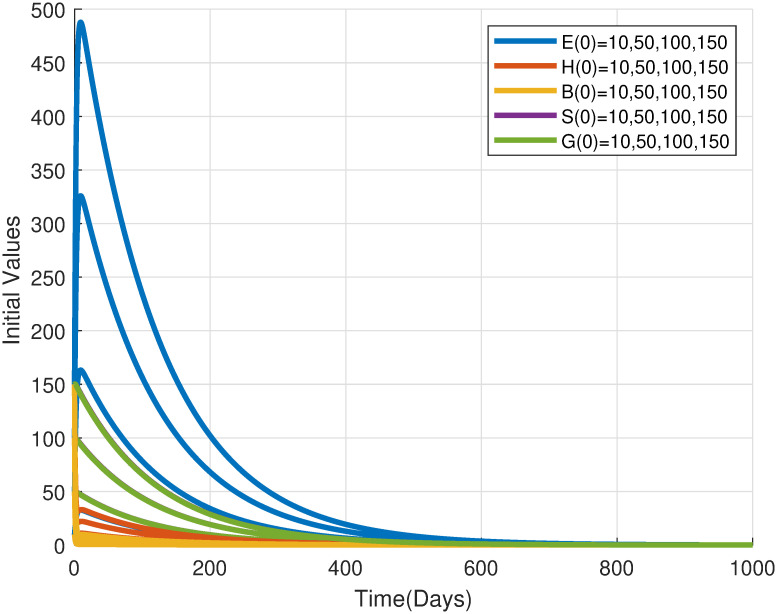
Time series of desert locust population densities for different initial conditions converging towards the extinction equilibrium. The autonomous model (14) was used with parameters from Table 2 with *ϕ* = 1.75, resulting in a basic offspring number $N_{0}=0.2636<1$.

As illustrated in Fig 9, the simulation results corroborate the theoretical prediction that a basic offspring number($N_{0}$) below unity drives the locust population towards extinction. This outcome aligns with the concept of an extinction trap, where the population dwindles due to insufficient reproductive success to offset mortality rates. Such conditions can arise from various factors, including unfavorable environmental conditions, limited food resources, or effective control measures. Understanding the factors influencing $N_{0}$ is crucial for developing sustainable pest management strategies aimed at preventing population collapse.

Building upon the analysis of the trivial equilibrium (*P*_0_), we now investigate the stability of the non-trivial equilibrium (P*), which represents a persistent locust population. By conducting numerical simulations, we aim to corroborate the theoretical prediction that P* is locally asymptotically stable when the basic offspring number ($N_{0}$) exceeds unity. Additionally, we will explore the system’s behavior under perturbations to assess the robustness of the non-trivial equilibrium.

**Fig 10:**
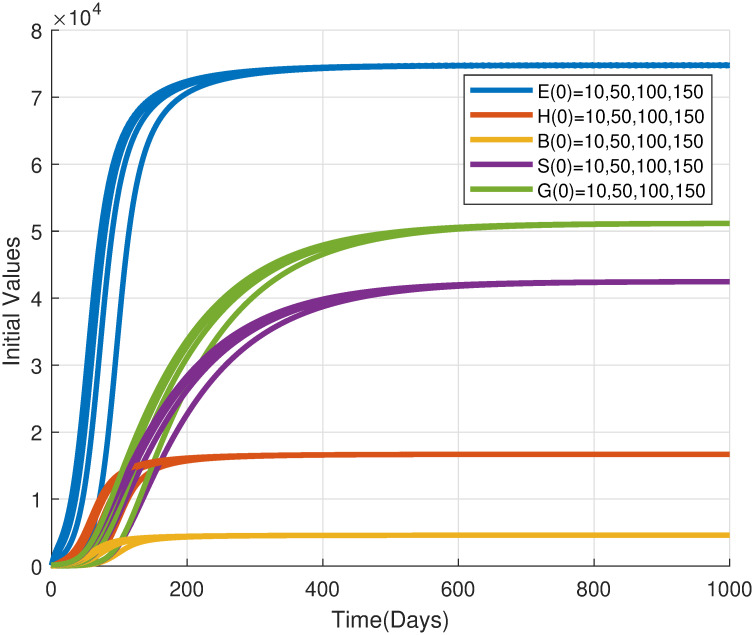
Time series of desert locust population densities (eggs, hoppers, bands, solitaries, and gregarious adults) converging towards the non-trivial equilibrium. The autonomous model (14) was used with parameters from Table 2 and *ϕ* = 25, *μ*_*h*_ = 0.25, *μ*_*b*_ = 0.3, resulting in a basic offspring number $N_{0}=16.2066>1$. Various initial conditions were utilized to illustrate the stability of the equilibrium.

Fig 10 illustrates the stability of the non-trivial equilibrium, where the locust population converges towards a persistent state defined by specific densities of eggs, hoppers, bands, solitaries, and gregarious adults. This equilibrium acts as an attractor, drawing the population towards it regardless of initial conditions. Understanding the factors influencing the stability of this equilibrium is crucial for developing effective locust management strategies, as it provides insights into the conditions that promote or suppress population growth.

#### 3.7.1 Temperature-dependent parameters effect

Sensitivity analysis identified egg-laying rate (*ϕ*), egg-hatching rate (*σ*), and hopper development rate (*γ*) as the primary parameters influencing desert locust population dynamics. To evaluate the impact of temperature-dependent variations in these parameters on the system (Eq (14)), numerical simulations were conducted.

Desert locust egg fecundity is significantly influenced by temperature. The temperature-dependent egg-laying rate, *ϕ*(*T*), defined by the function (7), captures this relationship. To quantify the impact of *ϕ*(*T*) on population dynamics, numerical simulations were conducted using the model described by system (4).

**Fig 11:**
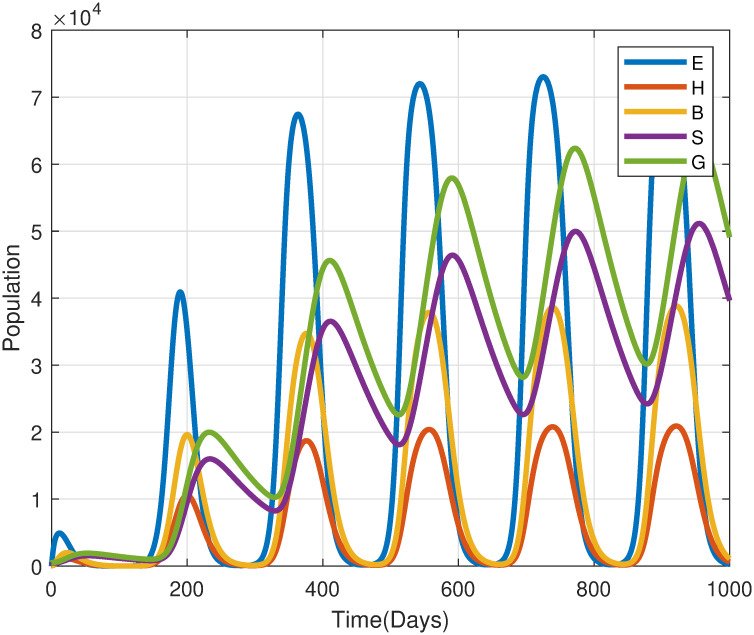
A simulation examining the effects of temperature-dependent fecundity rates(*ϕ*(*T*)) on the system (4).

Fig 11 illustrates the effect of temperature-dependent fecundity rates *ϕ*(*T*). The findings reveal that desert locust populations experience cyclic fluctuations driven by temperature dynamics. The egg population exhibits biannual peaks, occurring when temperatures reach optimal conditions for oviposition (*T*_opt_ ≈ 30°C). These peaks align with favorable environmental factors, such as the presence of moist sandy soils, which are critical for successful egg-laying and development. Conversely, deviations from optimal temperature conditions, either exceeding or falling below this threshold, lead to diminished egg-laying activity, consequently restricting population growth. These findings emphasize the significant role of temperature in influencing both the timing and intensity of locust population surges, with sensitivity to temperature during egg-laying being a crucial factor in outbreak dynamics.

Desert locust egg incubation typically lasts around two weeks but can vary significantly from 10 to 65 days due to fluctuating temperatures and other environmental factors. To assess the influence of temperature on egg hatching, numerical simulations employing the temperature-dependent egg hatching rate, *σ*(*T*) defined by the function (8), were conducted.

**Fig 12:**
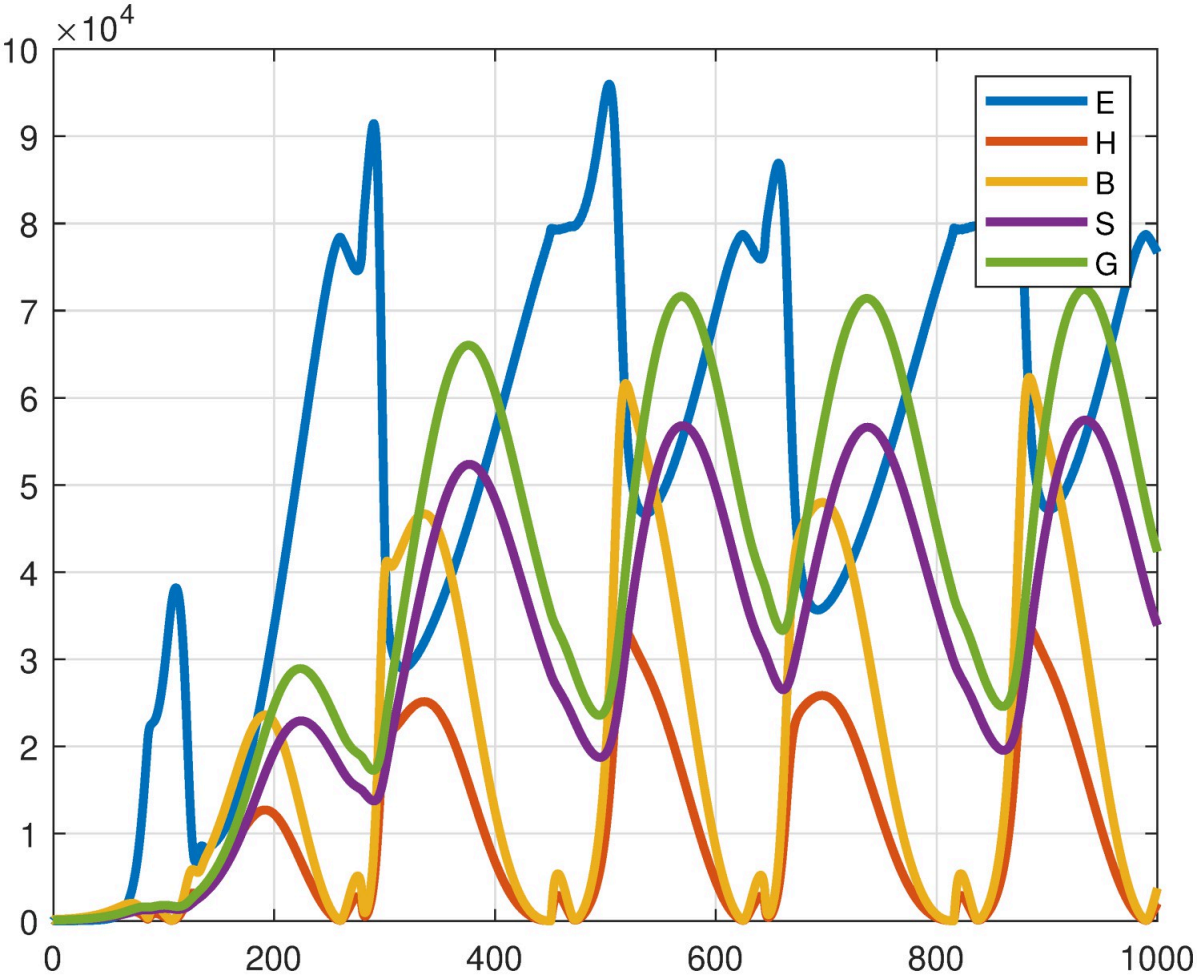
Simulated impact of temperature-dependent egg hatching rate *σ*(*T*) on desert locust population dynamics (4). Model parameters based on Table 2 with *ω* = 2, *T*_0_ = 20°C, *T*_1_ = 2°C, *κ* = 100.

Fig 12 illustrates the effect of temperature-dependent egg hatching rates *σ*(*T*), alongside other parameters at baseline values. Whereas temperature fluctuates, the egg hatching rates presented in section 3.1 induce cyclical fluctuations observable across various life stages. During periods of optimal temperatures (e.g., *T*_0_ = 25–35°C), egg-hatching rates increase, leading to synchronized population peaks. Conversely, extreme temperatures, whether excessively high or low, hinder this process by delaying hatching or increasing egg mortality, thereby suppressing population growth. This indicates that desert locust egg hatching is highly sensitive to temperature and requires an optimal temperature for successful hatching. It is observed that the failure in hatching due to temperature impacts also affects periodically all stages life cycle. Consequently, these findings underscore the importance of temperature-dependent hatching rates as a key regulatory factor in the population dynamics of desert locusts, influencing both the timing and magnitude of outbreaks.

Desert locust hoppers undergo five distinct developmental stages (instars) before transitioning to adulthood. Temperature significantly influences hopper development, with optimal growth rates occurring between 24°C to 32°C (Fig 12). To assess the impact of temperature-dependent hopper development on the overall locust population dynamics, simulations were conducted using the model (4).

**Fig 13:**
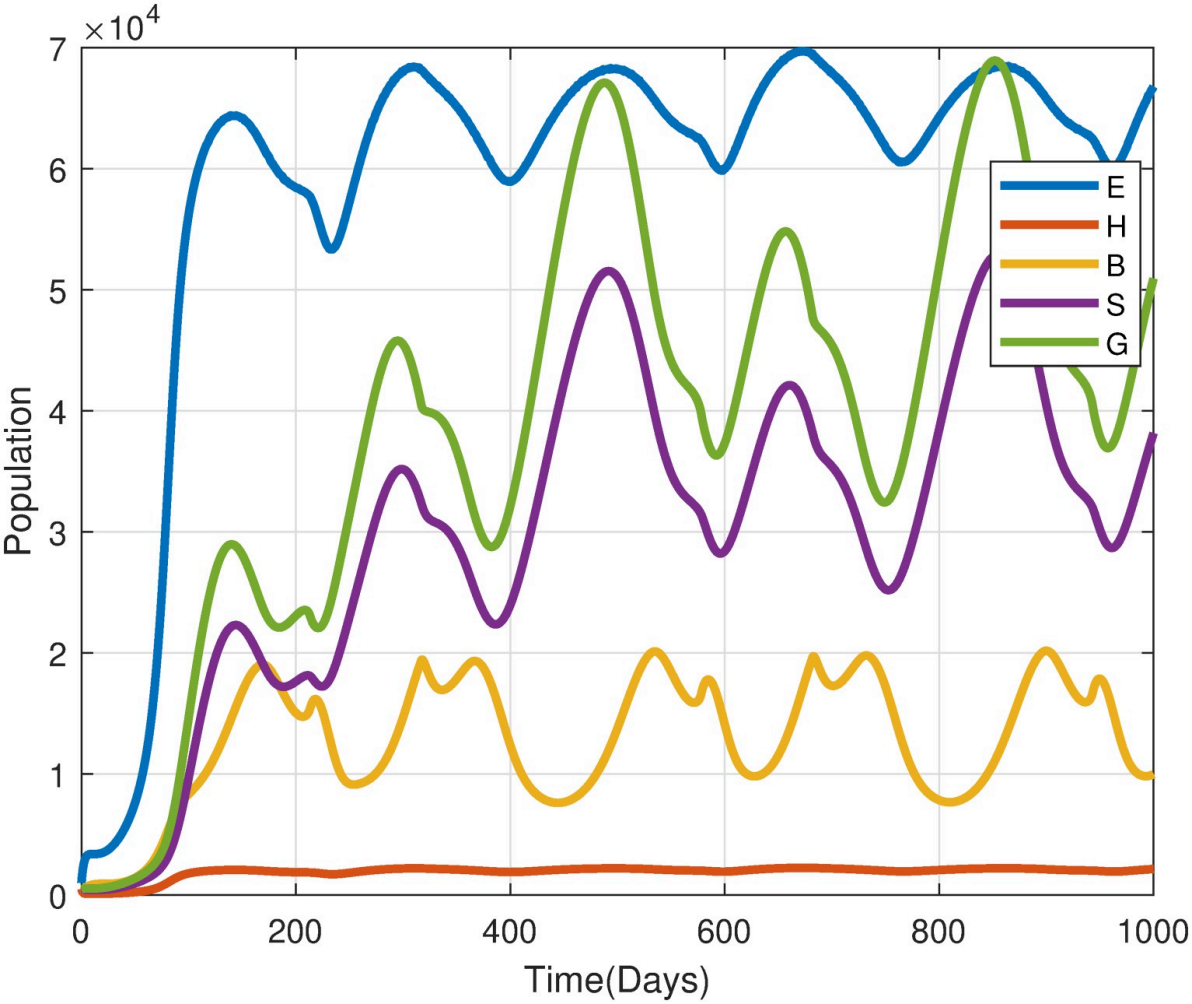
The impact of *γ*_*h*_(*T*).

**Fig 14:**
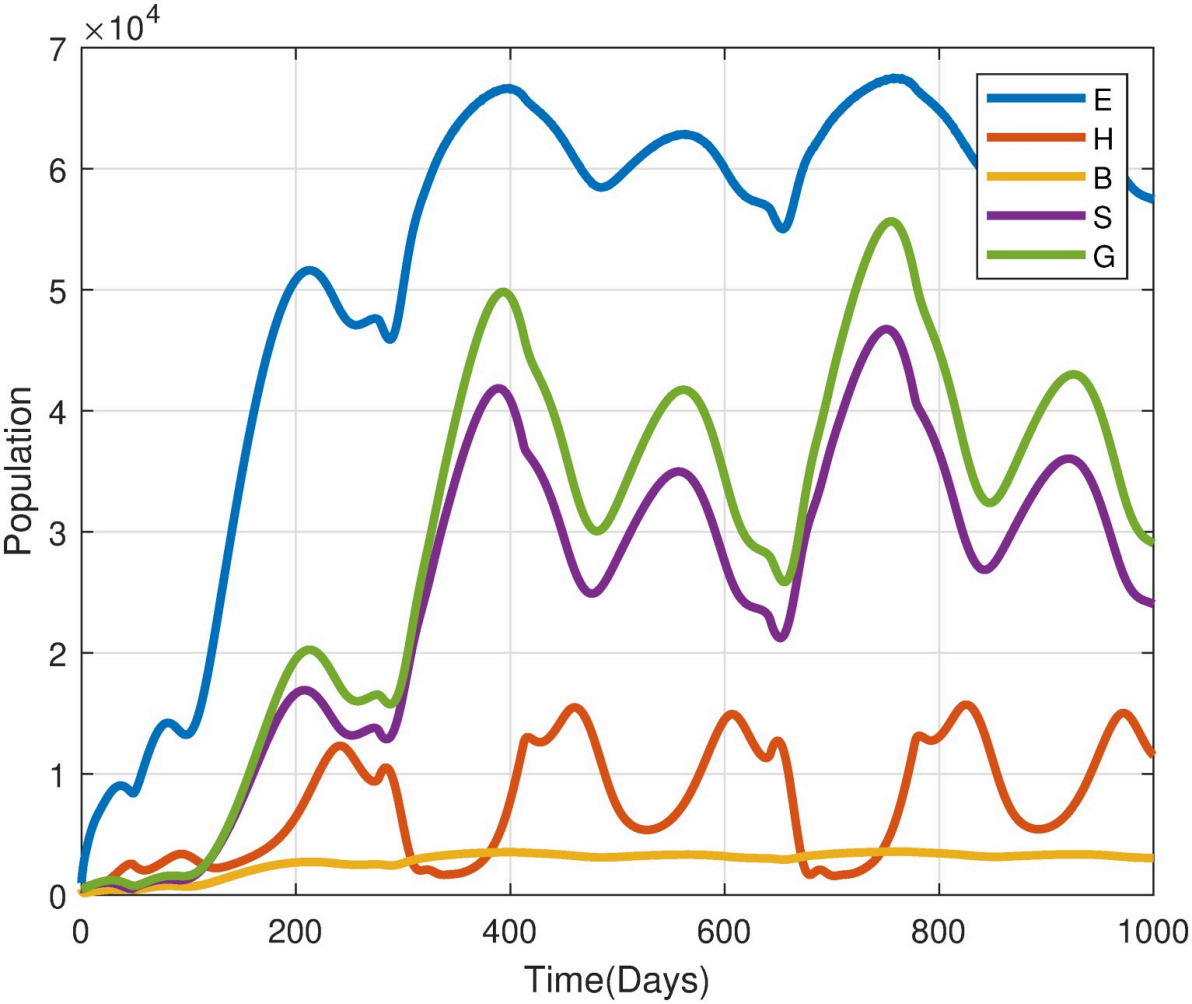
The impact of *γ*_*b*_(*T*).

The numerical simulations depicted in Figs 13 and 14 elucidate the ecological effects of temperature-dependent development rates on desert locust populations. Fig 13 investigates the effect of temperature on the solitarious hopper development rate (*γ*_*h*_(*T*)), whereas Fig 14 examines the influence of temperature on the band development rate (*γ*_*b*_(*T*)), with other parameters held constant. The outcomes indicate periodic fluctuations in population density across life stages, induced by variations in temperature. Optimal temperature ranges augment peak population growth, indicative of conducive conditions for development, reproduction, and phase transitions. Conversely, deviations from these optimal ranges result in a decline in population densities, implying ecological constraints imposed by suboptimal conditions. These findings highlight the critical role of temperature in modulating locust population dynamics, affecting their survival, growth, and the balance between solitarious and gregarious phases.

## 4 Discussion and conclusion

Desert locusts represent a significant threat to global agriculture and food security, thereby necessitating a detailed understanding of their population dynamics to aid in the development of predictive tools and control strategies [42]. This study introduces a mathematical model that investigates the influence of temperature on desert locust population dynamics, incorporating key life stages (egg, hopper, and adult) and behavioral phases (solitarious and gregarious). By integrating temperature-dependent parameters such as egg-laying, hatching, development, and mortality rates, the newly proposed model enhances the comprehension of how environmental factors impact locust population behaviors, extending insights from previous studies that examined insect population dynamics under varying climatic conditions [65, 67]. The theoretical analysis reveals complex dynamics, including the presence of multiple equilibria and backward bifurcations, which aligns with existing research highlighting the non-linear nature of population growth in locusts and other species under environmental variability [48, 67]. The derivation of the basic offspring number ($N_{0}$) serves as an essential entomological metric for evaluating population growth potential, and sensitivity analysis plays a critical role in measuring the correlation between model parameters and the offspring number. The temperature-dependent parameter analysis concurs with other research emphasizing the significance of temperature as a primary driver of population fluctuations in various insect species [68].

The simulation results demonstrate that temperature exerts a significant influence across all life stages of the desert locust. Moderate temperatures promote rapid development and increased survival, whereas extreme temperatures can impede growth by elevating mortality and diminishing fecundity. This sensitivity is particularly pronounced during the egg stage, where hatching success serves as a vital determinant of population growth. These findings are in agreement with previous studies that highlight the vulnerability of insect eggs to environmental variability [69]. Additionally, hopper development rates directly affect the timing of adult emergence and overall population dynamics, reinforcing empirical evidence that the speed of development is a critical factor in the locust life cycle [70]. While temperature is a pivotal driver, it is essential to consider other environmental and biological factors that influence locust outbreaks. Our model’s results are consistent with findings that underscore the significance of food availability, habitat heterogeneity, and social behavior in shaping locust aggregation and population growth. For example, research has demonstrated that dense, patchy vegetation promotes aggregation, whereas sparse, uniform plant cover leads to dispersal [71]. Consequently, future models must integrate these additional factors to more accurately predict the onset and spread of locust swarms, as environmental changes can substantially modify locust behavior [24]. The numerical simulations validate the theoretical predictions regarding equilibrium stability while revealing the potential for complex dynamics, including oscillatory behaviors and multiple equilibria under certain environmental conditions. These results underscore the challenges of predicting locust population behavior amid environmental variability, wherein even minor fluctuations in temperature or other factors can lead to significant shifts in population dynamics [38]. As with many ecological models, the precision of predictions relies heavily on accurate parameter estimation, highlighting the need for enhanced accuracy in temperature and other environmental inputs to optimize the model’s reliability.

In conclusion, this study underscores the substantial role temperature plays in influencing desert locust dynamics while highlighting the necessity for more comprehensive models that account for various environmental and biological factors. By enhancing our understanding of these dynamics, this research contributes to the development of more effective predictive tools for managing desert locust populations and mitigating the severe agricultural impacts they cause worldwide. Future research should prioritize expanding the model to incorporate additional complexities, such as spatial heterogeneity, rainfall fluctuation, and behavioral responses to environmental stimuli. Integrating these factors would provide a more comprehensive understanding of locust dynamics, thereby improving the model’s application for management strategies.
